# Microsatellite abundance across the Anthozoa and Hydrozoa in the phylum Cnidaria

**DOI:** 10.1186/1471-2164-15-939

**Published:** 2014-10-27

**Authors:** Dannise V Ruiz-Ramos, Iliana B Baums

**Affiliations:** Department of Biology, Pennsylvania State University, 208 Mueller Laboratory, University Park, PA 16802 USA

**Keywords:** Cnidaria, Microsatellites, Ancestral metazoan, Simple sequence repeats, Comparative genomics

## Abstract

**Background:**

Microsatellite loci have high mutation rates and thus are indicative of mutational processes within the genome. By concentrating on the symbiotic and aposymbiotic cnidarians, we investigated if microsatellite abundances follow a phylogenetic or ecological pattern. Individuals from eight species were shotgun sequenced using 454 GS-FLX Titanium technology. Sequences from the three available cnidarian genomes (*Nematostella vectensis*, *Hydra magnipapillata* and *Acropora digitifera*) were added to the analysis for a total of eleven species representing two classes, three subclasses and eight orders within the phylum Cnidaria.

**Results:**

Trinucleotide and tetranucleotide repeats were the most abundant motifs, followed by hexa- and dinucleotides. Pentanucleotides were the least abundant motif in the data set. Hierarchical clustering and log likelihood ratio tests revealed a weak relationship between phylogeny and microsatellite content. Further, comparisons between cnidaria harboring intracellular dinoflagellates and those that do not, show microsatellite coverage is higher in the latter group.

**Conclusions:**

Our results support previous studies that found tri- and tetranucleotides to be the most abundant motifs in invertebrates. Differences in microsatellite coverage and composition between symbiotic and non-symbiotic cnidaria suggest the presence/absence of dinoflagellates might place restrictions on the host genome.

**Electronic supplementary material:**

The online version of this article (doi:10.1186/1471-2164-15-939) contains supplementary material, which is available to authorized users.

## Background

**Figure 1:**
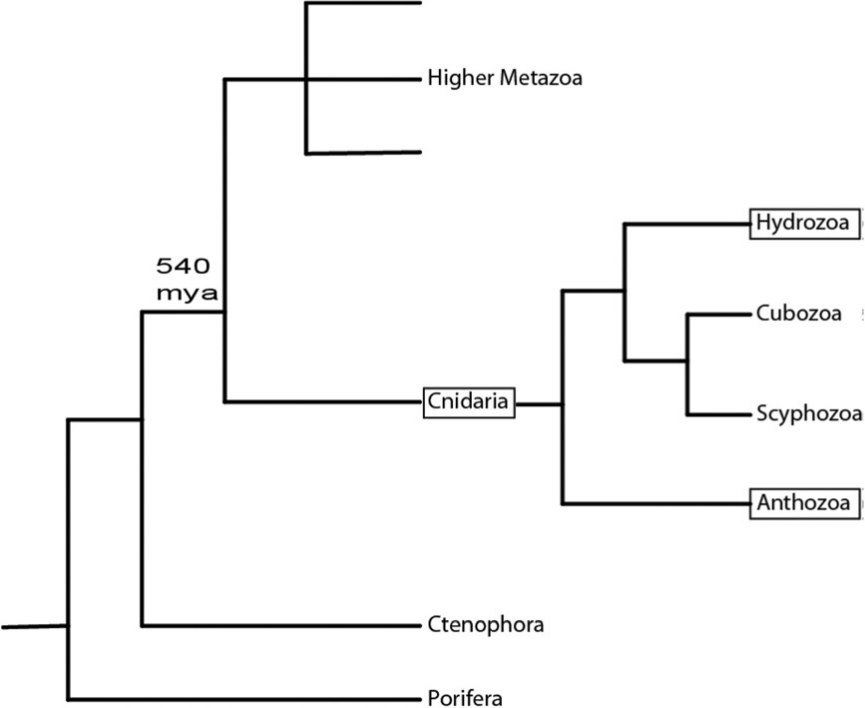
**Classical (non-molecular) phylogeny of the Metazoa adapted from Ball et al.**[1]**.** The split between the Cnidaria and the higher Metazoa occurred around 540 mya.

As ancestral metazoans [1] (Figure 1), Cnidarians provide an interesting system within which to study mutational processes. Cnidarians do not possess a sequestered germ line [2] and their members have a large diversity of life cycles, main adult stages (colonial or solitary), reproduction strategies (sexual, asexual, brooding, broadcasting) and morphological plasticity. Cnidarians also frequently harbor intra-cellular symbionts, the presence of which may, on the one hand, increase the amount of mutagens (reactive oxygen species, ROS) in host tissues [3], but on the other might exert pressure to limit genetic change, ensuring continued communication between partners [4]. Some of the differences in longevity, life cycle, morphology and symbiotic relationships across the Cnidaria might be related to differences in genomic composition or mutation rates [5]. For example, Cnidarians with short-lived medusa stages have faster mitochondrial mutation rates than cnidarians with long life spans and no medusa stages [6].

Microsatellites or Simple Sequence Repeats (SSRs) are sequences of no more than 6 basepairs (bp), repeated tandemly [7]. Point mutations and substitutions are responsible for producing proto-microsatellites (short sequence precursor of microsatellites). Once proteo-microsatellites are formed mutation rates will increase with increasing repeat length, until new mutations or substitutions stabilize mutation rates and the microsatellite sequence [8, 9]. DNA polymerase slippage and unequal recombination have been proposed to explain microsatellite expansion, and instability [10], while the frequency of microsatellites is associated with the presence of transposable elements [8, 11, 12].

Microsatellite loci are genomic features well suited for the study of mutational processes. Apart from high mutation rates, they influence a variety of phenotypic traits from skeletal morphology to host-pathogen interactions [13, 14]. Recent studies suggest that microsatellites play an important, direct role in genome evolution because microsatellites may affect DNA replication, translation and gene expression [13–15]. For example, in some disease-associated microsatellites, increases in repeat length can originate replication and chromosome rearrangement. In microsatellites located within coding or promoter regions, mutations in the repeats can modify patterns of gene expression.

Microsatellites have been found in every genome studied [7], and taxon-specific variation in microsatellite types and distribution are common [10]. Previous studies concluded that tetranucleotides are more abundant than trinucleotides in vertebrates, while di- and trinucleotides are more abundant in invertebrates [10, 16]. Some microsatellite features such as motif size and class can differ even among closely related species [10]; for example, extensive variation has been observed among *Drosophila* species [17]. The phylogenetic signal of microsatellites depends on the studied feature, for example, the relative abundance of AC and GC motifs and the average lengths of dinucleotides and trinucleotides appear to be conserved in most animals [17]. However, even when microsatellite composition is similar for closely related species, the relationship deteriorates as the evolutionary distance between species increases [18].

Comparisons of microsatellite abundances, lengths and repeat types within and among species have shed some light on the way genomes evolve [10, 12, 17]. Microsatellite types differ with their position in the genome, genome size does not correlate with the amount of microsatellites it contains, and mutation rates increase with microsatellite lengths. In Cnidaria, comparisons of the two sequenced genomes: *Nematostella vectensis* [19] and *Hydra magnipapillata* [20] showed noticeable differences in genome size, base composition, gene conservation and content of transposable elements; however, microsatellite abundance and frequency were not analyzed [5].

The objectives of this work were: 1) to describe the microsatellite abundance and composition in different members of the Cnidaria with focus on the Anthozoa and Hydrozoa, 2) to detect a phylogenetic signal in microsatellite abundance and composition and to 3) correlate the mitochondrial genome mutation rates and the presence of intra-cellular symbionts to microsatellite abundance and composition. We further provide resources for the design of microsatellite primers for future studies of cnidarian populations.

## Results

### Proportion of sequences with microsatellites

We partially sequenced the genomes of the following Cnidarians: black corals (*Leiopathes glaberrima* and *Tanacetipathes sp.*), candelabrum coral (*Eunicea flexuosa*), the octocoral *Plumarella sp.*, strawberry anemone (*Corynactis californica*), giant mushroom anemone (*Amplexidiscus fenestrafer*), plumose anemone (*Metridium senile*), and fire coral (*Millepora alcicornis*). A total of 929,398 sequences with a mean length of 474 bp and a mean trimmed sequence length of 192 bp were obtained from these eight cnidarian species. The sequences represent a quasi-random sample of a fraction of the eight species’ genomes. The overall proportion of sequences containing microsatellites varied among species, ranging from 1.3% in the octocoral *E. flexuosa* to 7.7% in the antipatharian *L. glaberrima* (Table 1).

**Table 1:** **Total number of sequences and percentage of sequences containing microsatellites and associated statistics**

Species	***Symbiodinium***present	Total sequences	Mean raw sequence length (bp)	Sequences with msats	% Sequences with msats	% Sequences with msats (after bootstrapped mean)	Bootstrapped mean of total msat	Bca CI (95%)	Total msat (count/Mbp)
*E. flexuosa* ^*s*^	Y	128422	452.9	1686	1.3	0.01	14.2	(7.13, 19.54)	85.3
*A. fenestrafer* ^*s*^	Y	132680	430.4	2169	1.6	0.01	17.2	(9.52, 28.43)	103.1
*M. alcicornis* ^*f*^	Y	114916	550.6	2081	1.8	0.02	19.5	(10.07, 43.78)	117.1
*Mean*		125339	478	1979^+^	1.6^#^	0.01	16.9^^^		
*L. glaberrima* ^*s*^	N	132680	418.9	10177	7.7	0.08	104.6	(58.30, 148.30)	627.9
*Tanacetipathes sp* ^*s*^	N	117973	470.2	6544	5.6	0.05	60.6	(31.25, 91.05)	363.7
*Plumarella sp* ^*s*^	N	108495	500.1	4594	4.2	0.05	51.3	(28.06, 71.87)	307.6
*C. californica* ^*s*^	N	100639	462.4	1797	1.8	0.02	17.6	(10.91, 24.65)	105.7
*M. senile* ^*s*^	N	109046	505.4	7519	6.9	0.08	88.6	(28.19, 176.72)	531.7
*Mean*		113767	471.4	6126	5.2	0.06	64.6		
N. vectensis* ^*s*^	Y	59149		3418	5.8	0.29	173.8	(98.60, 265.90)	1042.9
H. magnipapillata* ^*f*^	Y	126667		5438	4.3	0.22	277.7	(63.60, 868.30)	1666.1
A. digitifera* ^*s*^	Y	29765		225	0.8	0.68	201.8	(99.60, 446.30)	1210.7
*Mean*		71860		3027	3.6	0.40	217.8		1306.6
*Symbiodinium*									
* Clade C*		82331		12174					565.2
* A3*		94810		2674					154.9
* B1*		33816		19426					326.5
*Mean*		70319		11424					348.9

*Whole genome sequences downloaded from GenBank. Bootstrapped mean of % sequences with msats = the mean % of sequences with microsatellites calculated via bootstrapping (10000 iterations). Bootstrapped mean of total msat = the mean total count of microsatellites calculated via bootstrapping (10000 iterations). Bca CI: Bias corrected confidence interval calculated from 10000 bootstraps. *H. magnipapillata* and *M. alcicornis* have fast mutation rates (superscript ^f^), the remainder of the species has slow mutation rates (superscript ^s^). Symbiotic state is indicated by Y = yes and N = no. The symbiotic species have lower numbers of sequences with microsatellites^+^, percent of sequences with microsatellites^#^, and a lower bound for the bootstrapped mean of total microsatellites^^^ (t-tests, p <0.05, equal variances not assumed). Neither the total number of sequences not the mean raw sequence length differed between symbiotic and nonsymbiotic species (t-tests, p >0.05, equal variances not assumed).

DNA extracts from 3 of the species (Table 1) might have contained DNA from their intracellular symbionts, despite our efforts to isolate DNA from symbiont-free tissue (see methods). Thus, sequences containing microsatellites were aligned against a local database of *Symbiodinium* sequences. Sequences with more than 75% similarity to *Symbiodinium* were eliminated (if found). However, when aligning the putative *Symbiodinium* sequences against the full NCBI database the top hits were not to *Symbiodinium.* Regardless, putative *Symbiodinium* sequences were discarded from the analysis. The microsatellite cover was the same whether the putative *Symbiodinium* sequences were included or not. Microsatellite cover was also calculated for the *Symbiodinium* clades, their values being either similar (tetra- to hexanucleotides) or higher (di- and trinucleotides) than those of their hosts (Available in the Dryad Digital Repository: doi:10.5061/dryad.4k5st).

As a supplement to the eight species with partial genome sequences (PGS), scaffolds of whole genome sequences (WGS) of three other Cnidarians were processed as above, including WGS of *N. vectensis* (59,149 assembled scaffolds, with a mean length of 32,759 bp), *H. magnipapillata* (126,667 assembled scaffolds, mean length of 32,754 bp) and *A. digitifera* (29,765 assembled scaffolds, mean length 6,804 bp). The proportion of sequences containing microsatellites in the WGS species ranged from 0.8% in the scleractinian *A. digitifera* to 5.8% in the anemone *Nematostella* (Table 1).

### Summary statistics

**Figure 2:**
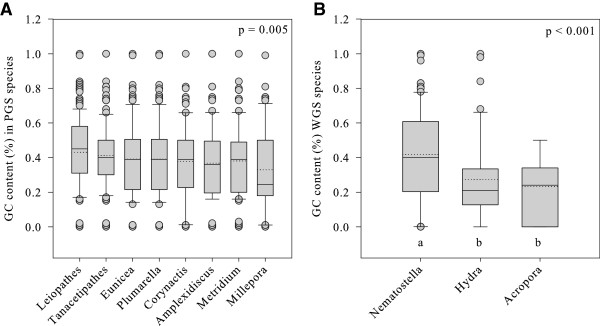
**GC content of microsatellite repeats found in the studied Cnidarian species (panel A and B).** PGS species include *Leiopathes* (n =628count/Mbp), *Tanacetipathes* (n =364), *Eunicea* (n =85), *Plumarella* (n =308), *Corynactis* (n =106), *Amplexidiscus* (n =103), *Metridium* (n =532), *Millepora* (n =117). Analysis of variance testing for differences in GC content among microsatellite repeat types in PGS was non-significant (p = 0.06). WGS species include *Nematostella* (n =1042), *Hydra* (n =1666), *A. digitifera* (n =1210). Analysis of variance testing for a difference in GC content among microsatellite repeats types in WGS was non-significant (p =0.10). The lowest boundary of the box indicates the 25^th^ percentile, the upper boundary indicates the 75^th^ percentile. Lines within the box indicate the mean (dashed line) and the median (solid line). Whiskers indicate the 10^th^ and 90^th^ percentiles; the filled circles are outliers.

Mean GC content ranged from 33% to 43% (Figure 2) in the 8 partial genome sequences (PGS) and from 23% to 42% in the WGS species, and differed between the PGS and the WGS (2 tailed t-test, p <0.001). GC content also differed among PGS species and among WGS species (Kruskal-Wallis One Way ANOVA, p <0.005, p <0.001).

**Figure 3:**
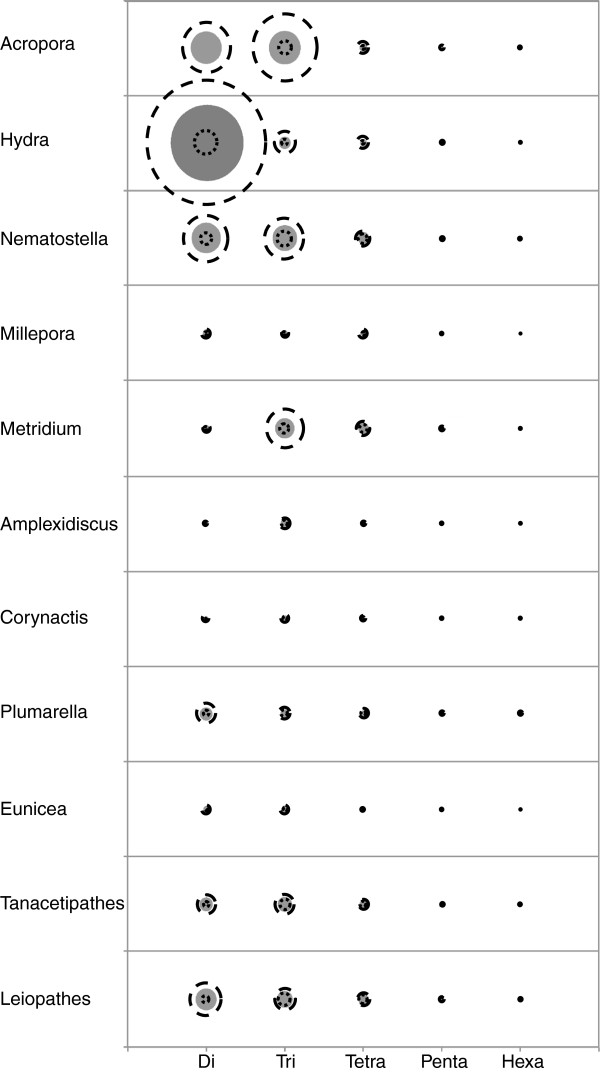
**Mean of microsatellites coverage found in the studied cnidarian species.** *Leiopathes* (n =628 count/Mbp), *Tanacetipathes* (n =364), *Eunicea* (n =85), *Plumarella* (n =308), *Corynactis* (n =106), *Amplexidiscus* (n =103), *Metridium* (n =532), *Millepora* (n =117), *Nematostella* (n =1047), *Hydra* (n =1666), *A. digitifera* (n =1210). The grey circles represent the proportion of microsatellites found. Dashed lines represent the upper and lower 95% confidence interval limits. If only one dashed circle is shown, the lowest C.I. =0.00. *Nematostella*, *Hydra* and *Acropora digitifera* microsatellites were derived from whole genome sequences while only a fraction of the genome was scanned for all other species (PGS). T-tests between coverage of a certain motive type between WGS and PGS were significant for mono- (p =0.01) and di- (p =0.01).

The observed microsatellite coverage was not significantly different between PGS and WGS species (t-test) when considering tri- (p =0.08), tetra- (p =0.21), penta- (p =0.37) and hexanucleotides (p =0.36), but differed for mono- (p =0.01) and dinucleotides (p =0.01) Figure 3, Table 2.

**Table 2:** **Microsatellite counts in the Cnidaria (by species)**

A)												
**Species**	**Mono-**	**Di-**	**BootMean**	**Bias**	**Bca CI (95%)**	**Tri-**	**BootMean**	**Bias**	**Bca CI (95%)**			
*Eunicea*	2	7.4	1.8	0.0	(0.3, 4.3)	23.1	2.3	0.0	(1.0, 4.3)			
*Amplexidiscus*	8	2.6	0.7	0.0	(0.1, 1.0)	39.1	3.9	0.0	(2.1, 6.2)			
*Millepora*	6	11.3	2.8	0.0	(0.6, 4.4)	16.5	1.6	0.0	(0.7, 3.0)			
*Leiopathes*	10	101	25.3	0.0	(2.7, 57.6)	144.9	14.5	0.1	(8.4, 25.2)			
*Tanacetipathes*	5	40	10	-0.1	(1.6, 19.8)	114.2	11.4	-0.1	(5.9, 21.4)			
*Plumarella*	4	36.7	9.2	0.0	(1.8, 21.6)	45.3	4.5	0.0	(2.5, 7.5)			
*Corynactis*	12	4.5	1.1	0.0	(0.2, 2.5)	22.1	2.2	0.0	(1.1, 3.6)			
*Metridium*	10	9	2.3	0.0	(0.8, 3.2)	215	21.5	0.0	(4.7, 81.2)			
*Nematostella*	138	198	49.5	-0.4	(7.3, 116.7)	353.7	35.4	0.0	(12.0, 90.4)			
*Hydra*	202	1249	312.3	1.3	(30.2, 837.1)	76.9	7.7	0.0	(2.0, 26.3)			
*Acropora*	42	231	55.8	-0.7	(0.0, 136.2)	594.0	59.4	0.3	(9.5, 243.7)			
**B)**												
**Species**	**Tetra-**	**BootMean**	**Bias**	**Bca CI (95%)**	**Penta-**	**BootMean**	**Bias**	**Bca CI (95%)**	**Hexa-**	**BootMean**	**Bias**	**Bca CI (95%)**
*Eunicea*	17.3	0.5	0.0	(0.3, 0.8)	23.1	0.2	0.0	(0.2, 0.3)	12.5	0.1	0.0	(0.0, 0.1)
*Amplexidiscus*	22.7	0.7	0.0	(0.4, 1.1)	17.4	0.2	0.0	(0.1, 0.3)	13.2	0.1	0.0	(0.0, 0.2)
*Millepora*	59.2	1.8	0.0	(0.8, 4.3)	16.3	0.2	0.0	(0.1, 0.3)	7.6	0.0	0.0	(0.0, 0.1)
*Leiopathes*	197.8	6	0.0	(4.1, 8.9)	85.5	1	0.0	(0.7, 1.4)	88.1	0.5	0.0	(0.4, 0.6)
*Tanacetipathes*	109.3	3.3	0.0	(2.0, 5.4)	46.4	0.5	0.0	(0.4, 0.7)	49	0.3	0.0	(0.2, 0.4)
*Plumarella*	82.2	2.5	0.0	(1.4, 4.9)	51.7	0.6	0.0	(0.4, 1.0)	87.3	0.5	0.0	(0.2, 1)
*Corynactis*	31.6	1	0.0	(0.6, 1.6)	20.9	0.2	0.0	(0.1, 0.3)	14.9	0.1	0.0	(0.0, 0.2)
*Metridium*	203.2	6.2	0.0	(3.4, 10.9)	66.6	0.7	0.0	(0.5, 1.2)	27.8	0.1	0.0	(0.1, 0.2)
*Nematostella*	243.9	7.3	0.0	(4.8, 12.1)	50.3	0.5	0.0	(0.3, 0.8)	59.1	0.2	0.0	(0.2, 0.4)
*Hydra*	83.9	2.5	0.0	(0.7, 7.8)	33.4	0.3	0.0	(0.1, 0.9)	20.9	0.1	0.0	(0.1, 0.2)
*Acropora*	151.3	3.2	0.0	(0.8, 7.6)	90.8	0.5	0.0	(0.0, 1.2)	102.2	0.2	0.0	(0.0, 0.5)

Mono-,di-, tri- (A) ,tetra-,penta- and hexa- (B) are microsatellite types. BootMean: the bootstrapped mean of the sample based on 10000 bootstraps. Bootstrapping is not appropriate for mononucleotide motifs. Bias: difference between the arithmetic mean and the bootstrapped sample. Bca CI (95%): the bias corrected accelerated interval percentile at the 95% confidence level as calculated via bootstrapping.

All microsatellite types (mono-, di-, tri-, tetra-, penta- and hexanucleotides) were found in the studied species, with the exception of *C. californica* in which mononucleotides were not detected. Of all possible motif combinations, 2 motifs of mono-, 4 di- (Table 3), 10 tri- (Table 4), 33 tetra- (Table 5), 77 penta- (Additional file 1: Table S1) and 160 types of hexanucleotides (Additional file 1: Table S2) were found in the sequenced data. Overall, trinucleotides and tetranucleotides were the most abundant types in Cnidaria, but noticeable differences were observed among species (Figure 3, Additional file 2).

**Table 3:** **Coverage (counts/Mbp) of dinucleotide motifs found in Cnidaria**

	***Symbiotic***			***Nonsymbiotic***					***WGS (Symbiotic)***			
	***Eunicea***	***Amplexidiscus***	***Millepora***	***Leiopathes***	***Tanacetipathes***	***Plumarella***	***Corynactis***	***Metridium***	***Nematostella***	***Hydra***	***Acropora***	***Mean***
**AC**	1.4	0.9	5.8	67.5	25.5	3.8	3.0	1.9	23.1	109.0	41.6	25.8
AG	0.7	1.2	2.4	28.2	11.1	6.2	1	3.1	29.1	57	0.0	14.0
**AT**	5.3	0.5	3.1	5.2	2.7	26.7	0.4	3.7	145.9	1079.8	181.6	132.3
CG	0.0	0.0	0.0	0.3	0.6	0.0	0.0	0.4	0.0	3.4	0.0	1.9

The two most abundant motifs are in bold.

**Table 4:** **Coverage (counts/Mbp) of trinucleotide motifs found in Cnidaria**

	***Leiopathes***	***Tanacetipathes***	***Eunicea***	***Plumarella***	***Corynactis***	***Amplexidiscus***	***Metridium***	***Millepora***	***Nematostella***	***Hydra***	***Acropora***	***Mean***
**AAC**	46.6	27.2	6.4	6.8	5.6	5.7	18.2	2.0	51.0	7.8	34.1	19.2
AAG	16.5	5.4	1.3	3.5	2.3	2.6	5.1	0.9	4.4	0.6	30.3	6.6
**AAT**	18.8	6.8	4.8	10.3	5.1	8.1	20.7	4.6	180.2	51.5	480.5	71.9
ACC	6	3.8	1	2.7	0.7	2.6	8.0	0.3	14.2	1.1	0.0	4.0
ACG	3.3	8.4	0.7	1.2	0.5	1.2	0.9	0.0	8.1	0.0	0.0	3.0
**ACT**	26.1	18.7	0.9	3.6	3.1	6.8	1.7	2.6	6.1	5.5	15.1	8.2
AGC	12.4	4.1	0.7	4.1	0.3	0.8	1.6	0.8	3.4	0.0	0.0	3.2
AGG	5.5	2.2	0.0	0.9	0.3	1	1.2	0.2	3.1	0.5	0.0	1.7
**ATC**	9.7	37.7	7.2	12.3	4.3	10	157.6	4.9	82.5	10.0	34.1	33.7
CCG	0.0	0.0	0.0	0.0	0.0	0.6	0.0	0.0	0.7	0.0	0.0	0.7

The most abundant motifs are in bold.

**Table 5:** **Coverage (counts/Mbp) of tetranucleotide motifs found in Cnidaria**

	***Leiopathes***	***Tanacetipathes***	***Eunicea***	***Plumarella***	***Corynactis***	***Amplexidiscus***	***Metridium***	***Millepora***	***Nematostella***	***Hydra***	***Acropora***	***Mean***
**AAAC**	29.8	13.6	1.6	5.7	3.1	1.8	36.6	1.8	18.8	2.7	22.7	12.6
AAAG	10.5	1.7	0.4	1.6	0.9	1.2	3.3	1.1	4.3	0.8	0.0	2.3
**AAAT**	11.0	4.5	2.0	6.1	5.3	3.6	12.3	5.0	46	19.4	26.5	12.9
AACC	4.5	2.5	0.6	1.2	0.3	0.1	6.6	0.0	1.9	0.0	0.0	1.6
AACG	2.3	0.7	0.1	0.8	0.3	0.0	4.4	0.0	1.9	0.0	0.0	1
AACT	2.8	0.8	0.7	1.5	0.5	0.1	0.9	0.2	4.3	1.2	0.0	1.2
AAGC	2.3	1.8	0.2	0.5	0.2	0.5	3.5	0.3	1.8	0.0	0.0	1.0
AAGG	8.9	3	0.1	4.3	0.4	0.7	3.7	0.3	1.2	0.0	0.0	2.1
AAGT	2.7	1.3	0.2	0.7	0.9	1.7	6.9	6.7	3.7	0.8	0.0	2.3
AATC	13.3	9.6	1.1	2.6	5.6	2.3	28.6	1.2	10.8	3.4	0.0	7.1
AATG	7.3	2.7	0.6	1.8	1.5	0.9	41.5	1	14.3	0.5	0.0	6.6
AATT	1.8	0.5	0.3	1	0.6	0.5	1.6	0.5	4.6	1.4	0.0	1.2
**ACAG**	19.8	15.8	0.7	18.3	0.6	1.0	12.8	2.1	28.5	0.8	0.0	9.1
ACCC	0.9	0.0	0.0	0.2	0.2	0.0	0.0	0.0	1.6	0.0	0.0	0.3
ACCG	0.7	0.8	0.2	0.0	0.2	0.0	0.8	0.3	4.6	0.0	0.0	0.7
ACCT	4.9	1.4	0.3	4.1	1	0.2	1	1.5	12.6	0.0	0.0	2.4
ACGC	8.3	3.1	0.1	0.0	0.2	0.2	0.0	0.2	0.9	0.0	0.0	1.2
ACGG	1	1.1	1.0	0.7	2.5	0.0	1	0.6	8.1	0.0	0.0	1.5
ACGT	0.7	0.7	0.1	0.0	0.0	0.0	0.3	0.3	0.0	0.0	0.0	0.2
ACTC	9.7	6.6	0.0	0.2	2.5	1.5	1.9	0.4	4	0.0	0.0	2.4
ACTG	7.1	10.2	1.1	0.5	0.6	1.5	6.8	1.4	4.7	0.0	0.0	3.1
AGCC	1.4	0.2	0.1	0.2	0.0	0.6	0.5	0.3	4.7	0.0	0.0	0.7
AGCG	1.2	0.3	0.2	0.2	0.0	0.0	0.0	0.0	0.0	0.0	0.0	0.2
AGCT	0.2	0.0	0.0	0.0	0.0	0.0	0.0	0.3	0.0	0.0	0.0	0.0
AGGC	2	0.7	0.4	0.9	0.0	0.0	0.7	0.2	8.1	0.0	0.0	1.2
AGGG	2.9	0.9	0.0	0.8	0.0	0.3	0.6	0.0	4.3	0.0	0.0	0.9
**ATAC**	20.7	18.4	1.8	7.2	1.3	2.2	2.3	10.2	7.8	45.0	37.8	14.1
**ATAG**	13.9	3.9	1.8	19.3	1.5	1.2	3.7	22.3	30.0	6	22.7	11.5
ATCC	4.2	2.1	0.2	1.1	0.8	0.2	20.3	0.7	8	0.0	0.0	3.4
ATCG	0.5	0.5	0.0	0.0	0.3	0.0	0.3	0.2	0.7	0.0	0.0	0.2
ATGC	0.6	0.0	0.4	0.6	0.0	0.0	0.6	0.0	0.0	0.5	0.0	0.2
CCCG	0.0	0.0	0.0	0.0	0.0	0.0	0.0	0.0	0.6	0.0	0.0	0.1
CCGG	0.0	0.0	0.8	0.0	0.0	0.0	0.0	0.0	0.0	0.0	0.0	0.1

The most abundant motifs are in bold.

**Figure 4:**
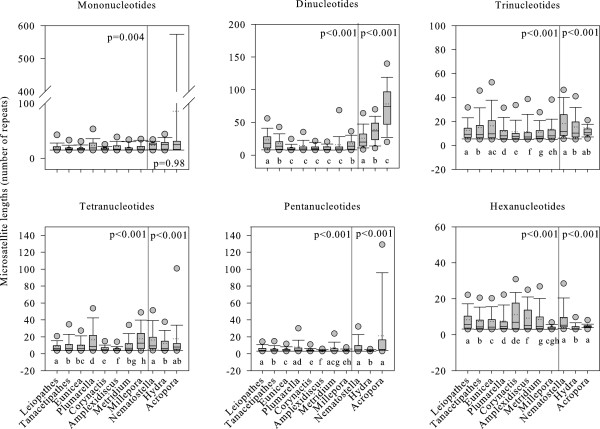
**Boxplots of the microsatellite repeat length (number of repeat copies) in the Cnidaria.** T-test between whole genome sequenced (WGS) and partial genome sequenced (PGS) species were significant (p <0.001) for di-, tri-, tetra-, hexa- and pentanucleotides (p <0.02). For PGS species, analyses of variance were significant (p <0.05) for tri-, tetra-, penta- and hexanucleotides. For the WGS species, analyses of variance were significant (p <0.001) for mono-, tri- and tetranucleotides. NS = not significant. Notice the varying scales of the y-axis. See Figure 1 for an explanation of the box plots.

The microsatellite lengths (number of repeat units) were also significantly different (One Way ANOVA, p <0.004) among species (Figure 4, Additional file 2). Mean repeat numbers ranged from 4 to 23 (repeats/microsatellite type) for the PGS species and between 4 and 86 repeats for the WGS species. However, sequences with long microsatellites (e.g. >23 repeats) were rare (mean of means =14.6 repeats, s.d. =5.4) in both WGS and PGS.

### Phylogeny and ecology

**Figure 5:**
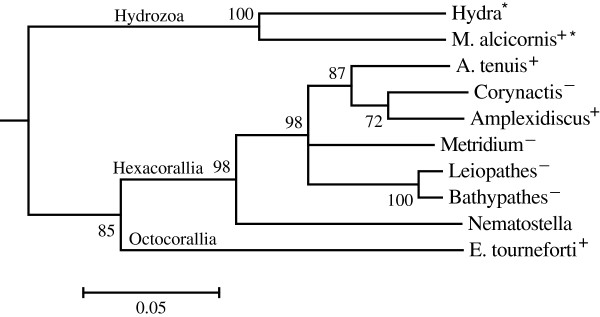
**Bayesian genealogy of ten cnidarian species studied, based on partial COI sequences (approx. 700 bp).** Posterior probabilities are shown next to the nodes. The scale bar represents 0.05% sequence divergence. Partial COI of *E. tourneforti* was used instead of *E. flexuosa*, *Bathypathes* instead of *Tanacetipathes*, and *A. tenuis* instead of *A. digitifera*.

A COI phylogeny of the studied species was constructed as a requirement to test evolutionary models of the microsatellite coverage, using a log-likelihood test. The COI tree showed the expected grouping of Hydrozoa, Octocorallia and Hexacorallia (Figure 5). *Nematostella vectensis* was basal to the Hexacorallia, but the long branch in *Nematostella* suggested increased levels of sequence divergence between *Nematostella* and the other species. The Corallimorpharia clustered with the Scleractinian sequences while the Antipatharia clustered with *M. senile* (Actiniaria). The phylogeny also suggested that the Actinaria (*N. vectensis*, *M. senile*) are paraphyletic.

The log-likelihood test between phylogeny and coverage of microsatellite types suggested abundance follows a random walk evolutionary model (Table 6). Regressions (p =1.00, Table 6) between phylogeny and coverage of microsatellite types were not significant, indicating there is no phylogenetic signal in the coverage and types of microsatellites. Regressions (p =1.00) between phylogeny and microsatellite lengths yielded similar results.

**Table 6:** **The relationship between phylogeny and microsatellite traits (performed with BayesTraits)**

	Model A	Model B	LRT (df =1)	p
mono	-55.10	-54.19	-1.82	1.00
di	-72.26	-72.15	-0.22	1.00
tri	-67.98	-67.98	0.00	1.00
tetra	-59.98	-59.94	-0.08	1.00
penta	-51.07	-50.98	-0.17	1.00
hexa	-52.40	-52.38	-0.04	1.00

Given are the log likelihoods for the microsatellites coverage assuming random walk evolution (Model A) and assuming directional evolution (Model B). LTR = Log likelihood test of Model A and Model B, p >0.05, model A is suggested.

**Figure 6:**
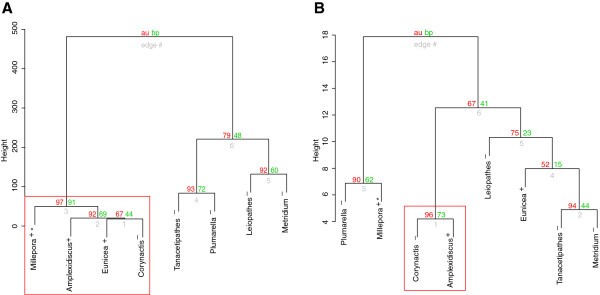
**Hierarchical cluster of cnidarian species: (A) microsatellite coverage, (B) mean repeat length.** Red box indicate groups with bootstrap values higher than 95%. BP (green) = Bootstrap values and AU (red) = approximate unbiased p-values. * Indicate species with fast mtDNA mutation rate. Symbiotic state is indicated with +/- symbols (+ symbiotic, - non-symbiotic). Clustering was performed on Euclidian distances using the Ward cluster method.

The hierarchical cluster analysis revealed a weak phylogenetic signal for the coverage and length of microsatellites (Figure 6). The significant clusters in each analysis had approximated unbiased p-values (AU) of >95% indicating that those clusters were supported by the data [21]. *Millepora alcicornis*, *E. flexuosa, A. fenestrafer* and *C. californica* grouped by microsatellite coverage (Figure 6a). Thus, clustering by microsatellite coverage discriminated between symbiotic and non-symbiotic corals (excepting *C. californica*). The corallimorpharians, *A. fenestrafer* and *C. californica* clustered by length of microsatellites (Figure 6b) making the corallimorpharia the only phylogenetic group recovered by the hierarchical clustering.

Non-symbiotic corals had about 6-fold higher microsatellite coverage than symbiotic corals (Mann–Whitney U Test, p =0.002, Additional file 1: Table S6a), but the microsatellites were similar in lengths (Mann–Whitney U Test, p =0.65, Additional file 1: Table S6b). Microsatellite coverage of *Symbiodinium* was higher than the microsatellite coverage found in their hosts (Kruskal-Wallis One Way ANOVA, p <0.001). Microsatellite coverage did not vary with rate of mitochondrial evolution (slow vs. fast) for microsatellite coverage (Mann–Whitney U Test, p =0.61, Additional file 1: Table S7a), and microsatellite length (Mann–Whitney U Test, p =0.99, Additional file 1: Table S7b).

## Discussion

Here, we described microsatellite abundances and lengths in the phylum Cnidaria with a focus on the Anthozoa and Hydrozoa. We investigated if these characters were predicted by phylogeny, mitochondrial mutation rates or ecology (presence of symbiotic algae). When clustering the Cnidarian species by microsatellite lengths and microsatellite coverage, only the two closely related corallimorpharian species grouped together; no phylogenetic signal was found in the clustering of the remaining 6 species representing higher taxonomic levels. A phylogenetic signal in microsatellite abundance and length in the Cnidaria thus appears to be lost fairly rapidly, but more data is needed to support this finding.

Interestingly, dinoflagellate-hosting Cnidaria had 6-fold lower microsatellite coverage and different microsatellite composition from those that do not host dinoflagellates. This result contrasted with the microsatellite coverage of their main associated dinoflagellates, which had also 5 to 6 orders of magnitude higher microsatellite coverage then their hosts. These observations prompted the hypothesis that the close association with dinoflagellates might place restrictions on microsatellite coverage and composition in Cnidarian genomes.

### Summary statistics

GC content is correlated with genomic features relevant in determining genome function like gene density, distribution of transposable elements and gene expression levels [22, 23]. Here, the average GC content in the microsatellite sequences varied between 23 and 43% (n =11 species). The GC content in the microsatellites did not differ among the studied species; thus the GC proportion in the microsatellites cannot explain differences in microsatellite abundance in the Cnidaria.

Microsatellite coverage in the partial genomes of eight species varied from 4.4 to 215, suggesting a wide range of microsatellite coverage for the phylum. These results mirror previous studies in which the densities of microsatellites vary within phyla [8, 10, 18, 24].

Microsatellites might influence genome evolution and, at the same time, the processes generating and maintaining microsatellites might be altered during the evolution of the genome [10, 25]. Clear signals in the abundance and type of microsatellite have been observed when comparing phyla and subphyla [10, 17, 24] and when comparing sister species [17], but microsatellite composition and coverage becomes more heterogeneous as the species divergence increases [18]. In this article, differences in the frequencies of microsatellite motif types were detected among the cnidarian species included. Frequency of microsatellite types was similar for some of the closely related species (*L. glaberrima* and *Tanacetipathes sp*; *C. californica* and *A. fenestrafer*), but differed for the other related group (Octocorallia).

All theoretically possible combinations of mono- (2), di- (4), tri- (10) and tetranucleotide (33) motifs were present in the PGS species. Eighty-nine of the 102 possible penta- and 172 of the 350 possible hexanucleotides were also observed. Pentameric and hexameric nucleotides generally have longer sequences [26], and thus we might not have been able to detect them because read lengths from *454* GS FXL Titanium had means of 192 bp after trimming.

Di-, tri- and tetranucleotides are the most abundant microsatellites in invertebrates [10, 16, 24], and this generally holds true for the Cnidaria. AC was the most frequent dinucleotide motif found in the studied species. This motif is also common in other metazoans, such as vertebrates and arthropods [10, 18]. CG motifs are rare in Cnidaria as well as in other metazoans [10, 16, 18]. In this dataset, AAT, ATC, AAC and ACT were the most abundant trinucleotides motifs. Interestingly, ACT was reported as rare in most of the previously sequenced vertebrate and invertebrate taxa [10], but is frequent in the Cnidaria. For example, ACT constitutes 40% of the trinucleotides observed in *Millepora* (Table 4).

Repeat number, length (bp) and motif type are indicators of microsatellite mutation rates [8, 11]. A greater number of repeats increases the probability of slippage and thus increases the mutability of the microsatellite [8, 11]. Mapping the location and identity of our microsatellites is not possible and, therefore, we cannot directly estimate mutation rate. However, based on the fact that mutability increases with microsatellite length [11] we suggest that the significant differences in average repeat lengths among cnidarian species (Figure 4) might indicate differing mutation rates among species. Further studies are required to test this hypothesis.

### Phylogenetic signal of microsatellite characteristics

Previous studies [10, 17] suggested that some microsatellite features (i.e. dominant microsatellite motif) might be related to the evolutionary history of the species and, therefore, those features should be concordant with the species’ phylogeny. To test this hypothesis in the Anthozoa and Hydrozoa, we performed hierarchical cluster analysis and Bayesian regressions on the proportion of microsatellite motifs and the microsatellite lengths.

The hierarchical cluster analyses suggested a weak phylogenetic signal for the coverage of microsatellite within the Cnidaria at the order level (Figure 6). Additional sub-family sampling is required before conclusions can be drawn about the similarity of microsatellite coverage among members of the same family. However, other studies suggest that increasing the number of species in a phylogenetic group usually increases the heterogeneity of the microsatellite composition and coverage [18].

The phylogenetic regression showed no relationship between microsatellite abundance or microsatellite length and phylogeny, suggesting evolution of these microsatellite characters was random. However, the dominance of di-, tri- and tetranucleotide motifs was shared between the Anthozoa and the Hydrozoa, the most distantly related Cnidarians [1, 5] (Figures 1, 3 and 5), suggesting that di-, tri- and tetranucleotide dominance might be the ancestral microsatellite state for Cnidaria. These results compare with the conclusions of Ross et al. [17] that the phylogenetic signal derived from microsatellite patterns depends on the microsatellite features under study.

### Symbiotic signal of microsatellite features

Members of the Cnidaria have diverse symbiotic relationships and this diversity might be related to differences in genomic composition or mutation rates [5]. We explored if, presence of intracellular algal symbionts in the genus *Symbiodinium*, and host mtDNA mutation rates were associated with microsatellite coverage and/or microsatellite length.

The symbiotic corals had 6-fold lower microsatellite coverage than the non-symbiotic corals. Similarly, the microsatellite coverage in *Symbiodinium* was up to 6-fold higher than the microsatellite coverage in their coral hosts. Therefore, difference in microsatellite coverage cannot be attributed to contamination by *Symbiodinium* sequences (Additional file 1: Table S3). The methods used for tissue collection and DNA extractions were biased against *Symbiodinium*, reducing the chances of overrepresentation of *Symbiodinium* sequences: DNA was extracted from *Symbiodinium*-free eggs (*Eunicea*), bleached colonies (*Millepora*), or the base of the anemone’s foot (*Amplexidiscus*). Thus the number of symbionts was expected to be low [27, 28]. The studied coral sequences were then aligned against sequences from various *Symbiodinium* Clades (provided by LaJeunesse et al., unpublished data) to remove potential contaminants. The frequency and type of microsatellites found in the only published *Symbiodinium* genome [29] was higher or similar to that of the studied corals. Therefore, we concluded that a potential contamination with *Symbiodinium* sequences would have increased microsatellite coverage, instead of decreasing it.

The intracellular nature of the symbiosis demands tight communication between the partners [30] and thus it is conceivable that the symbiotic state selects for stable host genomes. Alternatively, factors that co-vary with symbiosis state might explain the differences in microsatellite coverage. The symbiotic species included in this study occur in high temperature and light environments. High light and temperature combined with the mutation stress from reactive oxygen produced as a by-product of photosynthesis by their symbionts might select for efficient DNA repair mechanisms in these long-lived species. To further investigate if microsatellite coverage is influenced by symbionts or light/temperature stress, future work would benefit from the addition of a non-symbiotic tropical cnidarian (such as *Tubastrea coccinea*).

Previous studies suggested differences between nuclear and mitochondrial DNA mutation rates across the Cnidaria [31–33]. For example, the mitochondrial genomes of the Anthozoa have low mutation rates, while the mitochondrial genomes of Medusozoa (including the Hydrozoa) have mutation rates similar to more complex animals [32]. Slow mutation rates in the mitochondrial DNA point to efficient DNA repair mechanisms [31–33]; however, cnidarian nuclear allozymes and microsatellite markers show high heterozygosity indicative of high mutation rates [33–35].

We considered WGS and PGS species together to compare microsatellite abundances and lengths between Cnidaria with slow mtDNA mutation rates (*L. glaberrima*, *Tanacetipathes sp*, *Plumarella sp*, *C. californica*, *E. flexuosa*, *A. fenestrafer*, *M. senile*, *N. vectensis, A. digitifera*) and Cnidaria with fast mtDNA mutation rates (*M. alcicornis* and *H. magnipapillata*), and found no difference (p =0.61 for microsatellite coverage, Additional file 1: Table S7a; p =0.99 for microsatellite length, Additional file 1: Table S7b). While additional samples from the Meduzosoa and Hydrozoa (fast mtDNA mutation rates) would strengthen the comparison, our results lend some support to the idea that DNA repair is efficient in the Medusozoa and Hydrozoa but mitochondrion-specific.

## Conclusions

This study broadens our understanding of microsatellite distributions in non-model invertebrates. Almost every theoretically possible microsatellite motif was found. Trinucleotides and tetranucleotides were the most common microsatellites in the Cnidaria, challenging the convention that penta and hexanucleotides are most frequent in all nonvertebrate taxa [10].

Cnidarian species showed differences in microsatellite densities, between symbiotic and non-symbiotic cnidarians. Lower microsatellite coverage, although not lengths, in symbiotic corals suggest that either the symbiotic state itself or factors that co-vary with it, such as high light and high temperature, place restrictions on the host genome. Inclusion of non-symbiotic tropical Cnidarians will be informative in this regard.

## Methods

DNA was extracted using QIAGEN DNeasy kits with 2 elution steps of 5 min of elution time. High-quality DNA (~3 mg) was extracted from eight species: *Leiopathes glaberrima*, *Tanacetipathes sp*, *Corynactis californica*, *Amplexidiscus fenestrafer*, *Eunicea flexuosa Plumarella sp*, *Metridium senile* and *Millepora alcicornis* (Table 1). These samples encompass two classes (Anthozoa, Hydrozoa), three subclasses (Hexacorallia, Octocorallia, Hydroidolina) and five orders (Anthipatharia, Corallimorpharia, Alcyonacea, Actiniaria, and Anthoathecata) within the Cnidaria. Genomic libraries were prepared from the double-stranded DNA using Nextera DNA Sample Prep Kit (Epicentre Biotechnologies, Madison WI) and shotgun sequenced on a 454 GS-FLX sequencer using the Titanium Sequencing Kit (Roche Diagnostics Corporation, Indianapolis, IN).

Sequences were trimmed with PipeMeta [36] and assembled with the GS De Novo Assembler (Roche Diagnostics Corporation, Indianapolis, IN) keeping the default settings and a minimum sequence length of 45 base pairs. Sequences are available from NCBI Sequence read archive: *Leiopathes glaberrima* [Genbank: SRX323262], *Tanacetipathes* sp [Genbank: SRX327567], *Plumarella* sp [Genbank: SRX326898], *Eunicea flexuosa* [Genbank: SRX326897], *Corynactis californica* [Genbank: SRX326758], *Amplexidiscus fenestrafer* [Genbank: SRX326761], *Metridium senile* [Genbank: SRX327565], *Millepora alcicornis* [Genbank: SRX323169].

In addition, the whole genome sequence scaffolds from *Nematostella vectensis* [19], *Acropora digitifera* [37] and *Hydra magnipapillata* strain 105 [20] were obtained from GenBank. Whole genome sequences (WGS) were generated from symbiont-free tissues (larvae for *N. vectensis* and sperm for *A. digitifera*) [19, 37] except *Hydra* for which contaminant sequences were removed manually after assembly [19, 20, 37].

Several steps were taken to avoid/minimize sequence contamination with symbiotic dinoflagellate algae in the zooxanthellate corals (*E. flexuosa*, *A. fenestrafer* and *M. alcicornis*). When available, DNA was extracted from *Symbiodinium*-free larvae (*E. flexuosa*). *Amplexidiscus* DNA was extracted from the base of the anemone’s foot, which contains lower concentrations of symbionts [27]. *Millepora* DNA was extracted from bleached colonies which also feature a significantly reduced symbiont density [28]. In addition, the Partial Genome Sequences (those containing both flanking regions) were aligned to a custom database containing sequences from three *Symbiodinium* species: 454 sequences of clade C (Wham et al. unpublished) and assembled EST sequences of clades A and B [38], using BLASTn [39] and BLASTx [40] programs to check for the presence of *Symbiodinium* sequences. Sequences with more than 75 percent identity, alignment lengths larger than 50 bp and e-values lower than 1e^-05^ were filtered out of the cnidarian sequences because they represented putative *Symbiodinium* DNA and aligned against the NCBI database (Additional file 1: Table S3).

Cnidarian sequences were imported to the Tandem Repeat Finder (TRF) database [41] and processed using the default alignment parameters as follows: Match: 2; Mismatch: 7; Indels: 7. Sequences were categorized as having at least one flanking region or having two flanking regions (of at least 6 nucleotides) and run in the program SciRoKo [42] to extract all perfect tandem repeats between sizes two and six, containing at least three consecutive repeats. Microsatellite search parameters in SciRoKo were as follows: Search mode: Mismatched, Fixed Penalty; Mismatched Search Setting: Required score: 15; Mismatch penalty: 5; SSR seed minimum length: 8; SSR seed minimum repeat: 3, Maximum mismatches at once: 3. High error rate in homopolymer regions have been observed for Roche 454 [43]; for this reason mononucleotides sequences were excluded from the analyses.

Microsatellite coverage and GC content were calculated for each species based on the full data set, using SciRoKo [42]. Because only one representative of each species was sequenced, the coverage of microsatellite types for each species was bootstrapped using the boot function in R [44], to assign a measure of confidence to the coverage value. The subset of sequences with both flanking regions was used to calculate microsatellite length and repeat number. Analysis of Variance (ANOVA) was performed to compare microsatellite lengths among species using SPSS version 19.0 (IBM). Sequencing methodologies varied between species for which whole genomes are available (*N. vectensis* and *H. magnipapillata*: Sanger, *A. digitifera*: Roche 454GS-FLX and Illumina Genome Analyser IIx) and those that were sequenced in this study likely resulting in different sequencing biases between these two data sets [45]. Thus, WGS and PGS data sets were tested for differences due to sequencing methodology and were only combined when sequencing methodology did not influence the patterns.

For the phylogenetic analysis, COI sequences for each species were downloaded from Genbank (Additional file 1: Table S4), translated to proteins and aligned in Geneious version 5.5.4 [46]. Bayesian phylogenies were generated in Geneious with Mr. Bayes [47] using the mixed amino acid model with gamma distributed variation rates, a uniform branch length clock, and MCMC settings of 4 heated chains for 1000000 generations. A maximum clade credibility tree was constructed in TreeAnnotator v 1.6.2 in the BEAST package [48]. Regressions of the phylogeny and the microsatellite relative abundance and length were performed with BayesTraits [49] using Model A and B, and followed by a log likelihood test, to test for a relationship between phylogeny and microsatellite traits. Species were grouped based on microsatellite abundances and microsatellite lengths using hierarchical clustering in R, with the function hclust from the pvclust package [21].

### Availability of supporting data

Sequences are available at the NCBI Sequence Read Archive: SRX323262, SRX327567, SRX326898, SRX326897, SRX326758, SRX326761, SRX327565, SRX323169.

COI alignment and phylogenetic tree available from the Dryad Digital Repository: doi:10.5061/dryad.4k5st.

Microsatellite search results for the cnidarian and *Symbiodinium* species available from the Dryad Digital Repository: doi:10.5061/dryad.4k5st.

## Electronic supplementary material

Additional file 1: **Supporting tables.** **Table S1**: Proportion of pentanucleotides motifs found in Cnidaria. Most abundant motifs in bold.**Table S2**: Proportion of hexanucleotides motifs found in Cnidaria. Most abundant motifs in bold. **Table S3**: Results from the BLASTx alignments of Cnidarian sequences and *Symbiodinium* sequences. **Table S4**: Accession numbers of the sequences (700 bp) used to construct the Cytochrome Oxidase I genealogy. **Table S5**: Average microsatellite length (average nucleotide length/microsatellite type) found in Cnidaria. **Table S6**: Mann–Whitney Rank Sum Test between microsatellite cover (A) and microsatellite length (B) of symbiotic and non-symbiotic Cnidarians. **Table S7**: Mann–Whitney Rank Sum Test between microsatellite cover (A) and microsatellite length (B) for species with fast or slow mitochondrial evolution. (DOCX 225 KB)

Additional file 2: **Summary statistics.** (DOCX 121 KB)

## Abbreviations

SSRs: Simple Sequence Repeats
WGS: Whole genome sequenced
PGS: Partial genome sequenced
