# Supplementary material for: Microsatellite abundance across the Anthozoa and Hydrozoa in the phylum Cnidaria
Source: BMC Genomics. 2014 Oct 27;15(1):939. doi: 10.1186/1471-2164-15-939 (PMC4226868; doi:10.1186/1471-2164-15-939)
Supplement: Supplementary file 1 — Additional file 1: Supporting tables. Table S1: Proportion of pentanucleotides motifs found in Cnidaria. Most abundant motifs in bold.Table S2: Proportion of hexanucleotides motifs found in Cnidaria. Most abundant motifs in bold. Table S3: Results from the BLASTx alignments of Cnidarian sequences and Symbiodinium sequences. Table S4: Accession numbers of the sequences (700 bp) used to construct the Cytochrome Oxidase I genealogy. Table S5: Average microsatellite length (average nucleotide length/microsatellite type) found in Cnidaria. Table S6: Mann–Whitney Rank Sum Test between microsatellite cover (A) and microsatellite length (B) of symbiotic and non-symbiotic Cnidarians. Table S7: Mann–Whitney Rank Sum Test between microsatellite cover (A) and microsatellite length (B) for species with fast or slow mitochondrial evolution. (DOCX 225 KB) [file 12864_2013_6637_MOESM1_ESM.docx]

## Table S1 - Proportion of pentanucleotides motifs found in Cnidaria. Most abundant motifs in bold.

|  | *Leiopathes* | *Bathypathes* | *Eunicea* | *Plumarella* | *Corynactis* | *Amplexidiscus* | *Metridium* | *Millepora* | *Nematostella* | *Hydra* | *Acropora* | *Mean* |
| --- | --- | --- | --- | --- | --- | --- | --- | --- | --- | --- | --- | --- |
| **AAAAC** | 10.56 | 4.01 | 2.27 | 5.16 | 3.03 | 3.59 | 10.66 | 2.36 | 2.66 | 4.04 | 15.13 | 5.77 |
| **AAAAG** | 5.76 | 2.33 | 0.79 | 1.85 | 1.60 | 0.82 | 3.45 | 2.06 | 0.74 | 1.79 | 0.00 | 1.93 |
| **AAAAT** | 4.98 | 2.87 | 1.66 | 11.07 | 2.15 | 2.90 | 7.70 | 3.98 | 6.36 | 11.97 | 0.00 | 5.06 |
| AAACC | 0.32 | 0.20 | 0.22 | 0.16 | 0.00 | 0.00 | 0.54 | 0.00 | 0.00 | 0.00 | 0.00 | 0.13 |
| AAACG | 0.73 | 1.06 | 0.00 | 0.22 | 0.22 | 0.30 | 0.65 | 0.00 | 0.00 | 0.00 | 0.00 | 0.29 |
| AAACT | 0.78 | 1.80 | 1.01 | 1.09 | 0.00 | 0.30 | 1.35 | 0.29 | 0.74 | 0.00 | 0.00 | 0.67 |
| AAAGC | 0.64 | 0.41 | 0.39 | 0.16 | 0.17 | 0.00 | 0.65 | 0.00 | 0.00 | 0.00 | 0.00 | 0.22 |
| AAAGG | 0.73 | 0.00 | 0.17 | 0.81 | 0.00 | 0.39 | 1.18 | 0.00 | 0.00 | 0.00 | 0.00 | 0.30 |
| AAAGT | 0.37 | 0.45 | 0.00 | 0.98 | 0.00 | 0.22 | 1.35 | 0.29 | 0.00 | 0.60 | 0.00 | 0.39 |
| AAATC | 1.05 | 0.41 | 0.52 | 0.92 | 0.66 | 1.26 | 0.86 | 0.25 | 0.00 | 1.50 | 0.00 | 0.68 |
| AAATG | 0.82 | 0.49 | 0.44 | 1.09 | 0.77 | 0.61 | 1.08 | 0.54 | 0.00 | 0.00 | 0.00 | 0.53 |
| **AAATT** | 1.23 | 0.53 | 0.57 | 1.09 | 0.44 | 0.22 | 1.02 | 1.28 | 0.89 | 3.74 | 0.00 | 1.00 |
| AACAC | 3.02 | 2.25 | 0.39 | 0.98 | 0.66 | 0.00 | 1.13 | 0.00 | 0.74 | 0.00 | 0.00 | 0.83 |
| AACAG | 3.25 | 0.29 | 0.13 | 0.33 | 0.72 | 0.00 | 0.59 | 0.00 | 0.00 | 0.00 | 0.00 | 0.48 |
| AACAT | 1.55 | 0.33 | 0.17 | 0.87 | 0.17 | 0.13 | 0.81 | 0.44 | 1.18 | 0.00 | 0.00 | 0.51 |
| AACCC | 0.41 | 0.45 | 0.00 | 0.00 | 0.17 | 0.00 | 0.00 | 0.00 | 0.00 | 0.00 | 0.00 | 0.09 |
| AACCT | 0.46 | 0.12 | 0.00 | 0.27 | 0.22 | 0.00 | 0.00 | 0.00 | 0.00 | 0.00 | 0.00 | 0.10 |
| AACGC | 0.32 | 0.25 | 0.13 | 0.22 | 0.00 | 0.00 | 0.22 | 0.00 | 0.00 | 0.00 | 0.00 | 0.10 |
| AACGT | 0.37 | 0.20 | 0.00 | 0.22 | 0.17 | 0.00 | 0.27 | 0.00 | 0.00 | 0.00 | 0.00 | 0.11 |
| AACTC | 0.64 | 0.41 | 0.00 | 0.16 | 0.39 | 0.00 | 0.22 | 0.00 | 0.00 | 0.00 | 0.00 | 0.17 |
| AACTG | 0.32 | 0.53 | 0.61 | 0.16 | 0.28 | 0.26 | 0.00 | 0.00 | 0.00 | 0.00 | 0.00 | 0.20 |
| AACTT | 0.14 | 0.45 | 0.13 | 0.33 | 0.00 | 0.00 | 0.00 | 0.15 | 0.00 | 0.00 | 0.00 | 0.11 |
| AAGAC | 2.52 | 1.60 | 0.00 | 0.33 | 0.39 | 0.22 | 0.38 | 0.00 | 0.00 | 0.00 | 0.00 | 0.49 |
| AAGAG | 3.34 | 0.74 | 0.00 | 0.49 | 0.28 | 0.00 | 1.56 | 0.00 | 0.44 | 0.00 | 0.00 | 0.62 |
| AAGAT | 0.50 | 0.12 | 0.13 | 1.03 | 0.44 | 0.00 | 0.00 | 0.00 | 0.00 | 0.00 | 0.00 | 0.20 |
| AAGCC | 0.32 | 0.00 | 0.00 | 0.00 | 0.00 | 0.00 | 0.00 | 0.00 | 0.00 | 0.00 | 0.00 | 0.03 |
| AAGCG | 0.00 | 0.16 | 0.00 | 0.16 | 0.00 | 0.00 | 0.00 | 0.00 | 0.00 | 0.00 | 0.00 | 0.03 |
| AAGGC | 1.51 | 0.00 | 0.00 | 0.00 | 0.17 | 0.00 | 0.00 | 0.00 | 0.00 | 0.00 | 0.00 | 0.15 |
| AAGGG | 0.55 | 0.33 | 0.17 | 0.49 | 0.00 | 0.00 | 0.00 | 0.00 | 0.44 | 0.00 | 0.00 | 0.18 |
| AAGGT | 0.00 | 0.41 | 0.17 | 0.49 | 0.00 | 0.22 | 0.00 | 0.00 | 0.44 | 0.00 | 0.00 | 0.16 |
| AAGTC | 0.27 | 0.25 | 0.13 | 0.49 | 0.00 | 0.17 | 0.54 | 0.00 | 0.00 | 0.00 | 0.00 | 0.17 |
| AAGTG | 0.46 | 0.53 | 0.13 | 1.63 | 0.00 | 0.26 | 0.16 | 0.00 | 0.00 | 0.00 | 0.00 | 0.29 |
| **AATAC** | 3.61 | 3.15 | 1.57 | 1.03 | 0.55 | 0.95 | 3.72 | 0.15 | 7.69 | 0.90 | 15.13 | 3.50 |
| AATAG | 0.96 | 0.70 | 0.31 | 0.87 | 0.17 | 0.00 | 3.99 | 0.29 | 0.89 | 0.00 | 0.00 | 0.74 |
| **AATAT** | 0.41 | 0.33 | 0.52 | 2.01 | 0.33 | 0.00 | 2.05 | 0.54 | 1.92 | 5.23 | 15.13 | 2.59 |
| AATCC | 0.00 | 0.12 | 0.00 | 0.27 | 0.00 | 0.00 | 0.16 | 0.00 | 0.00 | 0.00 | 0.00 | 0.05 |
| AATCG | 0.00 | 0.00 | 0.00 | 0.00 | 0.00 | 0.00 | 0.16 | 0.00 | 0.00 | 0.00 | 0.00 | 0.01 |
| AATCT | 0.23 | 0.00 | 0.13 | 0.87 | 0.00 | 0.13 | 0.16 | 0.00 | 0.00 | 0.45 | 0.00 | 0.18 |
| AATGC | 0.23 | 0.16 | 0.35 | 0.60 | 0.17 | 0.13 | 0.27 | 0.00 | 0.00 | 0.00 | 0.00 | 0.17 |
| AATGG | 0.00 | 0.12 | 0.17 | 0.27 | 0.00 | 0.39 | 1.29 | 0.00 | 0.00 | 0.00 | 0.00 | 0.20 |
| AATGT | 1.05 | 1.47 | 0.31 | 0.81 | 0.66 | 0.30 | 1.72 | 0.39 | 0.59 | 0.00 | 0.00 | 0.66 |
| AATTC | 1.28 | 0.37 | 0.39 | 1.36 | 0.17 | 0.26 | 2.10 | 0.34 | 0.59 | 0.00 | 0.00 | 0.62 |
| ACACC | 2.65 | 0.74 | 0.48 | 0.33 | 0.33 | 0.00 | 1.13 | 0.00 | 0.00 | 0.00 | 0.00 | 0.51 |
| ACACG | 0.69 | 0.00 | 0.00 | 0.16 | 0.00 | 0.00 | 0.00 | 0.00 | 0.00 | 0.00 | 0.00 | 0.08 |
| ACACT | 1.42 | 1.35 | 0.00 | 0.60 | 0.00 | 0.17 | 0.81 | 0.00 | 0.00 | 0.00 | 0.00 | 0.40 |
| ACAGC | 2.88 | 1.72 | 0.00 | 0.00 | 0.00 | 0.00 | 0.70 | 0.00 | 0.00 | 0.00 | 0.00 | 0.48 |
| ACAGG | 0.55 | 0.12 | 0.17 | 0.00 | 0.00 | 0.00 | 0.00 | 0.00 | 0.00 | 0.00 | 0.00 | 0.08 |
| ACAGT | 0.59 | 0.53 | 0.17 | 0.38 | 0.33 | 0.17 | 0.27 | 0.00 | 2.81 | 0.00 | 0.00 | 0.48 |
| ACCAG | 0.14 | 0.41 | 0.00 | 0.00 | 0.00 | 0.00 | 0.00 | 0.00 | 0.00 | 0.00 | 0.00 | 0.05 |
| ACCCC | 0.78 | 0.25 | 0.13 | 0.43 | 0.50 | 0.35 | 0.38 | 0.20 | 2.37 | 0.00 | 0.00 | 0.49 |
| ACCCT | 0.00 | 0.00 | 0.13 | 0.00 | 0.00 | 0.00 | 0.00 | 0.00 | 1.48 | 0.00 | 0.00 | 0.15 |
| ACCGC | 0.23 | 0.00 | 0.00 | 0.00 | 0.00 | 0.00 | 0.00 | 0.00 | 0.00 | 0.00 | 0.00 | 0.02 |
| ACCTC | 0.00 | 0.00 | 0.00 | 0.60 | 0.00 | 0.00 | 0.00 | 0.00 | 0.00 | 0.00 | 0.00 | 0.05 |
| ACCGG | 0.00 | 0.00 | 0.00 | 0.00 | 0.00 | 0.00 | 0.00 | 0.00 | 0.44 | 0.00 | 0.00 | 0.04 |
| ACCGT | 0.00 | 0.00 | 0.00 | 0.00 | 0.00 | 0.00 | 0.22 | 0.00 | 0.00 | 0.00 | 0.00 | 0.02 |
| ACGAG | 0.23 | 0.49 | 0.00 | 0.16 | 0.00 | 0.00 | 0.00 | 0.00 | 0.00 | 0.00 | 0.00 | 0.08 |
| ACGCC | 0.00 | 0.16 | 0.00 | 0.00 | 0.00 | 0.00 | 0.00 | 0.00 | 0.00 | 0.00 | 0.00 | 0.01 |
| ACGCG | 0.14 | 0.00 | 0.00 | 0.00 | 0.00 | 0.00 | 0.00 | 0.00 | 0.00 | 0.00 | 0.00 | 0.01 |
| ACGGC | 1.46 | 0.00 | 0.00 | 0.00 | 0.00 | 0.00 | 0.00 | 0.00 | 0.00 | 0.00 | 0.00 | 0.13 |
| ACGTC | 0.64 | 0.12 | 0.44 | 0.54 | 0.00 | 0.26 | 0.00 | 0.00 | 0.00 | 0.00 | 0.00 | 0.18 |
| ACTAG | 0.00 | 0.12 | 0.00 | 0.00 | 0.28 | 0.00 | 0.27 | 0.00 | 0.00 | 0.00 | 0.00 | 0.06 |
| ACTCC | 4.44 | 0.57 | 0.00 | 0.00 | 0.00 | 0.00 | 0.00 | 0.00 | 0.00 | 0.00 | 0.00 | 0.46 |
| ACTCG | 0.14 | 0.20 | 0.00 | 0.00 | 0.00 | 0.00 | 0.00 | 0.00 | 0.00 | 0.00 | 0.00 | 0.03 |
| ACTCT | 0.69 | 0.86 | 0.00 | 0.60 | 0.00 | 0.00 | 0.27 | 0.00 | 0.00 | 0.00 | 0.00 | 0.22 |
| ACTGC | 0.73 | 0.37 | 0.00 | 0.22 | 0.00 | 0.00 | 0.00 | 0.00 | 0.00 | 0.00 | 0.00 | 0.12 |
| ACTGG | 0.69 | 0.37 | 1.14 | 0.00 | 0.17 | 0.00 | 0.81 | 0.00 | 0.44 | 0.00 | 0.00 | 0.33 |
| AGAGC | 1.10 | 0.94 | 0.00 | 0.00 | 0.00 | 0.00 | 0.48 | 0.00 | 0.00 | 0.00 | 0.00 | 0.23 |
| AGAGG | 1.33 | 0.25 | 0.00 | 0.16 | 0.00 | 0.00 | 0.38 | 0.00 | 0.44 | 0.00 | 0.00 | 0.23 |
| AGCCC | 0.14 | 0.16 | 0.00 | 0.00 | 0.00 | 0.00 | 0.00 | 0.00 | 0.00 | 0.00 | 0.00 | 0.03 |
| AGCCG | 0.00 | 0.00 | 0.00 | 0.00 | 0.00 | 0.00 | 0.16 | 0.00 | 0.00 | 0.00 | 0.00 | 0.01 |
| AGCCT | 0.00 | 0.20 | 0.00 | 0.00 | 0.00 | 0.00 | 0.00 | 0.00 | 0.00 | 0.00 | 0.00 | 0.02 |
| AGCGC | 0.14 | 0.00 | 0.00 | 0.00 | 0.00 | 0.00 | 0.00 | 0.00 | 0.00 | 0.00 | 0.00 | 0.01 |
| AGCTC | 0.14 | 0.00 | 0.13 | 0.00 | 0.00 | 0.00 | 0.00 | 0.00 | 0.00 | 0.00 | 0.00 | 0.02 |
| AGGCG | 0.00 | 0.00 | 0.00 | 0.00 | 0.00 | 0.00 | 0.00 | 0.00 | 1.18 | 0.00 | 0.00 | 0.11 |
| AGGGC | 0.27 | 0.12 | 0.00 | 0.00 | 0.00 | 0.00 | 0.00 | 0.00 | 0.00 | 0.00 | 0.00 | 0.04 |
| AGGGG | 1.69 | 0.66 | 0.17 | 0.33 | 0.44 | 0.52 | 1.29 | 0.00 | 4.58 | 0.00 | 0.00 | 0.88 |
| ATACC | 0.73 | 0.90 | 1.62 | 0.87 | 0.28 | 0.00 | 0.00 | 0.00 | 0.74 | 0.00 | 0.00 | 0.47 |
| ATACG | 0.37 | 0.12 | 0.26 | 0.00 | 0.00 | 0.17 | 0.00 | 0.00 | 0.00 | 0.00 | 0.00 | 0.08 |
| ATACT | 0.14 | 0.45 | 0.22 | 0.65 | 0.00 | 0.00 | 0.22 | 0.39 | 1.03 | 0.00 | 0.00 | 0.28 |
| ATAGC | 0.37 | 0.25 | 0.00 | 0.16 | 0.00 | 0.00 | 0.16 | 0.00 | 0.00 | 0.00 | 0.00 | 0.09 |
| ATAGG | 0.00 | 0.12 | 0.00 | 0.33 | 0.00 | 0.00 | 0.48 | 0.00 | 0.00 | 0.00 | 0.00 | 0.08 |
| ATATC | 0.69 | 0.29 | 0.31 | 0.92 | 0.00 | 0.13 | 0.70 | 0.00 | 0.00 | 0.00 | 0.00 | 0.28 |
| ATCAC | 0.82 | 0.74 | 0.92 | 0.33 | 0.28 | 0.00 | 2.48 | 0.00 | 0.44 | 0.00 | 0.00 | 0.55 |
| ATCCC | 0.00 | 0.33 | 0.00 | 0.38 | 0.00 | 0.00 | 0.22 | 0.00 | 0.59 | 0.00 | 0.00 | 0.14 |
| ATCCG | 0.00 | 0.00 | 0.17 | 0.27 | 0.17 | 0.00 | 0.00 | 0.00 | 0.00 | 0.00 | 0.00 | 0.06 |
| ATCGC | 0.18 | 0.37 | 0.00 | 0.00 | 0.00 | 0.00 | 0.00 | 0.00 | 0.00 | 0.00 | 0.00 | 0.05 |
| ATCTC | 0.23 | 0.33 | 0.00 | 0.49 | 0.22 | 0.00 | 0.27 | 0.00 | 0.00 | 0.00 | 0.00 | 0.14 |
| ATGAC | 0.59 | 0.12 | 0.13 | 0.27 | 0.00 | 0.00 | 0.97 | 0.00 | 0.74 | 0.00 | 0.00 | 0.26 |
| ATGCC | 0.64 | 0.00 | 0.74 | 0.00 | 0.00 | 0.00 | 0.00 | 0.20 | 0.00 | 0.00 | 0.00 | 0.14 |

## Table S2 - Proportion of hexanucleotides motifs found in Cnidaria. Most abundant motifs in bold.

|  | *Leiopathes* | *Bathypathes* | *Eunicea* | *Plumarella* | *Corynactis* | *Amplexidiscus* | *Metridium* | *Millepora* | *Nematostella* | *Hydra* | *Acropora* | *Mean* |
| --- | --- | --- | --- | --- | --- | --- | --- | --- | --- | --- | --- | --- |
| **AAAAAC** | 2.88 | 0.57 | 0.00 | 0.76 | 0.39 | 0.65 | 0.75 | 0.69 | 0.44 | 3.74 | 0.00 | 0.99 |
| AAAAAG | 1.37 | 0.53 | 0.00 | 0.43 | 0.33 | 0.00 | 0.16 | 0.54 | 0.00 | 0.00 | 0.00 | 0.31 |
| **AAAAAT** | 2.52 | 0.37 | 0.17 | 10.37 | 0.39 | 0.22 | 0.48 | 0.69 | 0.89 | 3.29 | 0.00 | 1.76 |
| AAAACC | 0.00 | 0.20 | 0.00 | 0.22 | 0.00 | 0.00 | 0.00 | 0.00 | 0.00 | 0.00 | 0.00 | 0.04 |
| AAAACG | 0.37 | 0.12 | 0.00 | 0.16 | 0.00 | 0.17 | 0.00 | 0.00 | 0.00 | 0.00 | 0.00 | 0.07 |
| AAAACT | 0.91 | 0.00 | 0.22 | 0.27 | 0.00 | 0.00 | 0.16 | 0.00 | 0.00 | 0.00 | 0.00 | 0.14 |
| AAAAGC | 0.32 | 0.00 | 0.00 | 0.27 | 0.00 | 0.00 | 0.00 | 0.00 | 0.00 | 0.00 | 0.00 | 0.05 |
| AAAAGG | 0.00 | 0.25 | 0.00 | 0.27 | 0.17 | 0.00 | 0.00 | 0.00 | 0.00 | 0.00 | 0.00 | 0.06 |
| AAAAGT | 0.00 | 0.12 | 0.00 | 0.16 | 0.00 | 0.00 | 0.00 | 0.00 | 0.00 | 0.00 | 0.00 | 0.03 |
| AAAATC | 0.14 | 0.25 | 0.00 | 0.27 | 0.00 | 0.13 | 0.32 | 0.00 | 0.00 | 0.45 | 0.00 | 0.14 |
| AAAATG | 0.46 | 0.33 | 0.00 | 0.22 | 0.00 | 0.13 | 0.22 | 0.34 | 0.00 | 0.45 | 0.00 | 0.20 |
| AAAATT | 0.55 | 0.16 | 0.13 | 0.33 | 0.39 | 0.00 | 0.54 | 0.00 | 0.59 | 0.90 | 0.00 | 0.33 |
| AAACAC | 1.88 | 0.78 | 0.13 | 0.38 | 0.00 | 0.00 | 0.00 | 0.00 | 0.00 | 0.00 | 0.00 | 0.29 |
| **AAACAG** | 0.27 | 0.00 | 0.00 | 16.34 | 0.00 | 0.00 | 0.00 | 0.00 | 0.00 | 0.00 | 0.00 | 1.51 |
| AAACAT | 0.14 | 0.12 | 0.00 | 0.22 | 0.00 | 0.00 | 0.00 | 0.00 | 0.00 | 0.60 | 0.00 | 0.10 |
| AAACCT | 0.32 | 0.45 | 0.00 | 0.16 | 0.00 | 0.00 | 0.00 | 0.00 | 0.00 | 0.00 | 0.00 | 0.08 |
| **AAACGC** | 0.37 | 0.00 | 0.00 | 0.43 | 0.00 | 0.00 | 0.00 | 0.00 | 2.51 | 0.00 | 11.35 | 1.33 |
| AAACGT | 0.00 | 0.00 | 0.00 | 0.00 | 0.00 | 0.00 | 0.00 | 0.00 | 0.44 | 0.00 | 0.00 | 0.04 |
| AAACTG | 0.14 | 0.12 | 0.00 | 0.33 | 0.00 | 0.00 | 0.00 | 0.00 | 0.00 | 0.00 | 0.00 | 0.05 |
| AAAGAC | 1.01 | 0.25 | 0.00 | 0.33 | 0.00 | 0.00 | 0.00 | 0.00 | 0.00 | 0.00 | 0.00 | 0.14 |
| AAAGAG | 0.23 | 0.45 | 0.00 | 0.33 | 0.00 | 0.00 | 0.00 | 0.00 | 0.00 | 0.00 | 0.00 | 0.09 |
| AAAGGG | 0.00 | 0.00 | 0.00 | 0.00 | 0.00 | 0.00 | 0.00 | 0.00 | 0.44 | 0.00 | 0.00 | 0.04 |
| AAAGGT | 0.00 | 0.00 | 0.00 | 0.00 | 0.00 | 0.17 | 0.00 | 0.00 | 0.00 | 0.00 | 0.00 | 0.02 |
| AAAGTG | 0.00 | 0.00 | 0.13 | 0.27 | 0.00 | 0.00 | 0.00 | 0.00 | 0.00 | 0.00 | 0.00 | 0.04 |
| AAAGAT | 0.00 | 0.12 | 0.00 | 0.38 | 0.00 | 0.00 | 0.00 | 0.00 | 0.00 | 0.00 | 0.00 | 0.05 |
| AAAGTT | 0.27 | 0.00 | 0.00 | 0.27 | 0.00 | 0.00 | 0.00 | 0.00 | 0.00 | 0.00 | 0.00 | 0.05 |
| **AAATAC** | 0.14 | 0.16 | 0.00 | 11.51 | 0.00 | 0.17 | 0.32 | 0.00 | 0.00 | 0.00 | 0.00 | 1.12 |
| AAATAG | 0.00 | 0.00 | 0.00 | 0.00 | 0.00 | 0.00 | 0.16 | 0.00 | 0.00 | 0.00 | 0.00 | 0.01 |
| AAATAT | 0.18 | 0.00 | 0.00 | 0.16 | 0.00 | 0.00 | 0.43 | 0.00 | 0.89 | 1.35 | 0.00 | 0.27 |
| AAATCT | 0.14 | 0.57 | 0.00 | 0.16 | 0.00 | 0.00 | 0.16 | 0.00 | 0.00 | 0.00 | 0.00 | 0.09 |
| AAATGC | 0.00 | 0.00 | 0.00 | 0.00 | 0.00 | 0.00 | 0.16 | 0.00 | 0.00 | 0.00 | 0.00 | 0.01 |
| AAATGT | 0.00 | 0.12 | 0.00 | 0.22 | 0.00 | 0.00 | 0.16 | 0.00 | 0.00 | 0.00 | 0.00 | 0.05 |
| AAATTC | 0.32 | 0.00 | 0.00 | 0.27 | 0.00 | 0.00 | 0.16 | 0.00 | 0.00 | 0.00 | 0.00 | 0.07 |
| AAATTG | 0.23 | 0.00 | 0.00 | 0.16 | 0.00 | 0.00 | 0.16 | 0.15 | 0.00 | 0.00 | 0.00 | 0.06 |
| AAATTT | 0.00 | 0.00 | 0.00 | 0.00 | 0.00 | 0.00 | 0.00 | 0.15 | 0.00 | 0.00 | 0.00 | 0.01 |
| AACAAG | 2.52 | 2.54 | 0.22 | 0.49 | 0.00 | 0.00 | 0.38 | 0.00 | 0.00 | 0.00 | 0.00 | 0.56 |
| AACACC | 1.01 | 0.20 | 0.00 | 0.22 | 0.00 | 0.00 | 0.43 | 0.00 | 0.00 | 0.00 | 0.00 | 0.17 |
| AACACG | 0.59 | 0.00 | 0.00 | 0.49 | 0.22 | 0.00 | 0.00 | 0.00 | 0.00 | 0.00 | 0.00 | 0.12 |
| AACACT | 0.18 | 0.00 | 0.00 | 0.16 | 0.00 | 0.00 | 0.00 | 0.00 | 0.00 | 0.00 | 0.00 | 0.03 |
| AACAGC | 3.80 | 0.33 | 0.00 | 0.43 | 0.00 | 0.00 | 0.00 | 0.00 | 1.03 | 0.00 | 0.00 | 0.51 |
| AACAGG | 0.50 | 0.25 | 0.00 | 0.22 | 0.00 | 0.00 | 0.00 | 0.00 | 0.00 | 0.00 | 0.00 | 0.09 |
| AACAGT | 0.27 | 0.00 | 0.00 | 0.16 | 0.00 | 0.00 | 0.00 | 0.00 | 0.44 | 0.00 | 0.00 | 0.08 |
| **AACATC** | 0.37 | 0.53 | 0.17 | 18.19 | 0.17 | 0.00 | 0.16 | 0.00 | 0.00 | 0.00 | 0.00 | 1.78 |
| AACATG | 0.14 | 0.25 | 0.00 | 0.33 | 0.00 | 0.00 | 0.00 | 0.00 | 0.00 | 0.00 | 0.00 | 0.07 |
| AACCAC | 0.41 | 0.00 | 0.26 | 0.54 | 0.00 | 0.00 | 0.27 | 0.00 | 0.00 | 0.00 | 0.00 | 0.13 |
| AACCAT | 0.00 | 0.20 | 0.00 | 0.16 | 0.00 | 0.00 | 0.16 | 0.00 | 0.00 | 0.00 | 0.00 | 0.05 |
| AACCAG | 0.14 | 0.00 | 0.00 | 0.38 | 0.00 | 0.00 | 0.00 | 0.00 | 0.00 | 0.00 | 0.00 | 0.05 |
| AACCCC | 0.00 | 0.00 | 0.00 | 0.00 | 0.00 | 0.00 | 0.00 | 0.00 | 1.18 | 0.00 | 0.00 | 0.11 |
| **AACCCT** | 2.38 | 3.52 | 1.31 | 0.43 | 6.44 | 4.42 | 2.80 | 0.34 | 6.36 | 0.90 | 15.13 | 4.00 |
| AACCGT | 0.00 | 0.12 | 0.00 | 0.16 | 0.00 | 0.00 | 0.00 | 0.00 | 0.00 | 0.00 | 0.00 | 0.03 |
| AACGAC | 0.87 | 0.41 | 0.87 | 0.27 | 0.00 | 0.00 | 0.00 | 0.00 | 0.00 | 0.00 | 0.00 | 0.22 |
| AACGAG | 0.41 | 0.00 | 0.00 | 0.22 | 0.00 | 0.00 | 0.00 | 0.00 | 0.00 | 0.00 | 0.00 | 0.06 |
| AACGAT | 0.14 | 0.37 | 0.00 | 0.16 | 0.00 | 0.00 | 0.59 | 0.00 | 0.00 | 0.00 | 0.00 | 0.11 |
| AACGCC | 0.23 | 0.00 | 0.00 | 0.43 | 0.00 | 0.00 | 0.00 | 0.00 | 0.00 | 0.00 | 0.00 | 0.06 |
| AACGGC | 0.14 | 0.12 | 0.00 | 1.19 | 0.00 | 0.17 | 0.00 | 0.00 | 0.00 | 0.00 | 0.00 | 0.15 |
| AACGGT | 0.14 | 0.00 | 0.00 | 0.16 | 0.00 | 0.00 | 0.00 | 0.00 | 0.00 | 0.00 | 0.00 | 0.03 |
| AACTAC | 0.14 | 0.37 | 0.00 | 0.43 | 0.00 | 0.00 | 0.27 | 0.00 | 0.00 | 0.00 | 0.00 | 0.11 |
| AACTAT | 0.14 | 0.00 | 0.00 | 0.22 | 0.00 | 0.00 | 0.00 | 0.00 | 0.00 | 0.00 | 0.00 | 0.03 |
| AACTCG | 0.00 | 0.53 | 0.00 | 0.27 | 0.00 | 0.00 | 0.00 | 0.00 | 0.00 | 0.00 | 0.00 | 0.07 |
| AACTGC | 0.00 | 0.20 | 0.13 | 0.54 | 0.00 | 0.00 | 0.00 | 0.00 | 0.00 | 0.00 | 0.00 | 0.08 |
| AACTGT | 0.00 | 0.00 | 0.31 | 4.40 | 0.00 | 0.00 | 0.00 | 0.00 | 0.00 | 0.00 | 0.00 | 0.43 |
| AACTTC | 0.18 | 0.00 | 0.00 | 0.27 | 0.00 | 0.00 | 0.00 | 0.00 | 0.00 | 0.00 | 0.00 | 0.04 |
| AACTTG | 0.23 | 0.12 | 0.00 | 0.16 | 0.00 | 0.13 | 0.27 | 0.00 | 0.00 | 0.00 | 0.00 | 0.08 |
| AAGACC | 0.27 | 0.00 | 0.00 | 1.57 | 0.00 | 0.00 | 0.00 | 0.00 | 0.00 | 0.00 | 0.00 | 0.17 |
| AAGACG | 0.27 | 0.00 | 0.00 | 0.60 | 0.00 | 0.00 | 0.00 | 0.00 | 0.00 | 0.00 | 0.00 | 0.08 |
| AAGACT | 0.23 | 0.00 | 0.00 | 0.76 | 0.00 | 0.00 | 0.00 | 0.00 | 0.00 | 0.00 | 0.00 | 0.09 |
| AAGAGC | 1.37 | 1.68 | 0.00 | 0.38 | 0.00 | 0.00 | 0.00 | 0.00 | 0.00 | 0.00 | 0.00 | 0.31 |
| AAGAGG | 1.69 | 0.74 | 0.00 | 0.16 | 0.00 | 0.00 | 0.00 | 0.00 | 0.00 | 0.00 | 0.00 | 0.24 |
| AAGATG | 0.18 | 0.25 | 0.00 | 0.43 | 0.00 | 0.26 | 0.00 | 0.15 | 0.00 | 0.00 | 0.00 | 0.12 |
| AAGCAC | 3.52 | 1.31 | 0.00 | 0.16 | 0.00 | 0.43 | 0.16 | 0.00 | 0.00 | 0.00 | 0.00 | 0.51 |
| AAGCAG | 0.82 | 0.00 | 0.00 | 0.33 | 0.00 | 0.00 | 0.00 | 0.00 | 0.00 | 0.00 | 0.00 | 0.10 |
| AAGCAT | 0.50 | 1.88 | 0.00 | 0.00 | 0.39 | 0.00 | 0.32 | 0.00 | 0.00 | 0.00 | 0.00 | 0.28 |
| AAGCTT | 0.00 | 0.00 | 0.00 | 0.00 | 0.22 | 0.00 | 0.00 | 0.00 | 0.00 | 0.00 | 0.00 | 0.02 |
| AAGCCC | 0.14 | 0.00 | 0.00 | 0.00 | 0.00 | 0.00 | 0.00 | 0.00 | 0.00 | 0.00 | 0.00 | 0.01 |
| AAGCGC | 0.00 | 0.33 | 0.00 | 0.00 | 0.00 | 0.00 | 0.22 | 0.00 | 0.00 | 0.00 | 0.00 | 0.05 |
| AAGGAC | 1.42 | 6.47 | 0.00 | 0.00 | 0.00 | 0.00 | 0.00 | 0.00 | 0.00 | 0.00 | 0.00 | 0.72 |
| AAGGAG | 4.07 | 0.49 | 0.00 | 0.00 | 0.00 | 0.00 | 0.97 | 0.00 | 0.00 | 0.00 | 0.00 | 0.50 |
| AAGGCC | 0.00 | 0.00 | 0.00 | 0.00 | 0.00 | 0.00 | 0.00 | 0.00 | 0.00 | 0.45 | 0.00 | 0.04 |
| AAGGGC | 0.37 | 0.12 | 0.00 | 0.00 | 0.00 | 0.00 | 0.00 | 0.00 | 0.00 | 0.00 | 0.00 | 0.04 |
| AAGGGG | 0.14 | 0.00 | 0.00 | 0.00 | 0.00 | 0.00 | 0.00 | 0.00 | 0.00 | 0.00 | 0.00 | 0.01 |
| AAGGGT | 0.00 | 0.00 | 0.00 | 0.00 | 0.33 | 0.00 | 0.00 | 0.00 | 0.00 | 0.00 | 0.00 | 0.03 |
| AAGGTC | 0.00 | 0.00 | 0.00 | 0.00 | 0.00 | 0.22 | 0.00 | 0.00 | 0.00 | 0.00 | 0.00 | 0.02 |
| AAGTAC | 0.18 | 0.12 | 0.00 | 0.00 | 0.00 | 0.00 | 0.00 | 0.00 | 0.00 | 0.00 | 0.00 | 0.03 |
| AAGTAG | 0.00 | 0.00 | 0.00 | 0.00 | 0.00 | 0.00 | 0.00 | 0.00 | 1.48 | 0.00 | 0.00 | 0.13 |
| AAGTAT | 0.00 | 0.00 | 1.14 | 0.00 | 0.00 | 0.00 | 0.00 | 0.00 | 0.00 | 0.00 | 0.00 | 0.10 |
| AAGTGC | 0.00 | 0.25 | 0.00 | 0.00 | 0.00 | 0.00 | 0.00 | 0.00 | 0.00 | 0.00 | 0.00 | 0.02 |
| AATAAC | 0.64 | 0.49 | 0.31 | 0.00 | 0.00 | 0.00 | 1.02 | 0.00 | 1.92 | 0.45 | 0.00 | 0.44 |
| AATAAG | 0.55 | 0.16 | 0.00 | 0.00 | 0.00 | 0.00 | 0.22 | 0.00 | 0.00 | 0.00 | 0.00 | 0.08 |
| AATACC | 0.00 | 0.16 | 0.00 | 0.00 | 0.00 | 0.00 | 0.00 | 0.00 | 0.00 | 0.00 | 0.00 | 0.01 |
| AATACT | 0.18 | 0.20 | 0.00 | 0.00 | 0.00 | 0.00 | 0.00 | 0.00 | 0.00 | 0.00 | 0.00 | 0.03 |
| AATAGC | 0.46 | 0.16 | 0.00 | 0.00 | 0.00 | 0.00 | 0.00 | 0.00 | 1.33 | 0.00 | 0.00 | 0.18 |
| AATAGT | 0.46 | 0.00 | 0.00 | 0.00 | 0.00 | 0.00 | 0.00 | 0.00 | 2.66 | 0.00 | 0.00 | 0.28 |
| AATATC | 0.00 | 0.00 | 0.00 | 0.00 | 0.00 | 0.00 | 0.00 | 0.29 | 0.00 | 0.00 | 0.00 | 0.03 |
| AATATG | 0.00 | 0.00 | 0.00 | 0.00 | 0.00 | 0.13 | 0.00 | 0.00 | 0.00 | 0.00 | 0.00 | 0.01 |
| AATATT | 0.00 | 0.00 | 0.00 | 0.00 | 0.00 | 0.00 | 0.38 | 0.00 | 0.89 | 0.00 | 0.00 | 0.12 |
| AATCAC | 0.23 | 0.12 | 0.00 | 0.00 | 0.00 | 0.00 | 0.16 | 0.00 | 0.00 | 0.00 | 0.00 | 0.05 |
| AATCAG | 0.00 | 0.12 | 0.17 | 0.00 | 0.00 | 0.00 | 0.00 | 0.00 | 0.00 | 0.00 | 0.00 | 0.03 |
| AATCAT | 0.55 | 0.20 | 0.44 | 0.00 | 0.00 | 0.00 | 0.38 | 0.00 | 0.59 | 0.00 | 0.00 | 0.20 |
| AATCCT | 0.00 | 0.20 | 0.00 | 0.00 | 0.00 | 0.00 | 0.00 | 0.00 | 0.00 | 0.00 | 0.00 | 0.02 |
| AATCTC | 0.14 | 0.49 | 0.00 | 0.00 | 0.00 | 0.00 | 0.00 | 0.00 | 0.00 | 0.00 | 0.00 | 0.06 |
| AATCTG | 0.27 | 0.00 | 0.00 | 0.00 | 0.00 | 0.00 | 0.00 | 0.00 | 0.00 | 0.00 | 0.00 | 0.02 |
| AATGAC | 0.37 | 0.20 | 0.00 | 0.00 | 0.00 | 0.13 | 0.48 | 0.00 | 1.18 | 0.00 | 0.00 | 0.21 |
| AATGAG | 0.27 | 0.00 | 0.00 | 0.00 | 0.00 | 0.00 | 0.00 | 0.00 | 0.00 | 0.00 | 0.00 | 0.02 |
| AATGAT | 0.87 | 0.57 | 0.00 | 0.00 | 0.00 | 0.22 | 2.53 | 0.54 | 2.51 | 0.00 | 0.00 | 0.66 |
| AATGGC | 0.23 | 0.00 | 0.00 | 0.00 | 0.00 | 0.00 | 0.00 | 0.00 | 0.00 | 0.00 | 0.00 | 0.02 |
| AATGGG | 0.00 | 0.16 | 0.00 | 0.00 | 0.00 | 0.00 | 0.00 | 0.00 | 0.00 | 0.00 | 0.00 | 0.01 |
| AATGGT | 0.00 | 0.00 | 0.00 | 0.00 | 0.66 | 0.52 | 0.16 | 0.00 | 0.00 | 0.00 | 0.00 | 0.12 |
| AATGTG | 0.18 | 0.16 | 0.00 | 0.00 | 0.00 | 0.00 | 0.00 | 0.00 | 0.00 | 0.00 | 0.00 | 0.03 |
| AATTAC | 0.00 | 0.00 | 0.31 | 0.00 | 0.00 | 0.00 | 0.00 | 0.00 | 0.00 | 0.00 | 0.00 | 0.03 |
| ACACAG | 1.01 | 0.82 | 0.00 | 0.00 | 0.00 | 0.00 | 0.00 | 0.00 | 0.00 | 0.00 | 0.00 | 0.17 |
| ACACCC | 0.18 | 0.00 | 0.00 | 0.00 | 0.00 | 0.00 | 0.00 | 0.00 | 0.00 | 0.00 | 0.00 | 0.02 |
| ACACCT | 0.14 | 0.00 | 0.00 | 0.00 | 0.00 | 0.00 | 0.00 | 0.00 | 0.00 | 0.00 | 0.00 | 0.01 |
| ACACGC | 1.60 | 0.61 | 0.00 | 0.00 | 0.00 | 0.00 | 0.00 | 0.00 | 0.00 | 0.00 | 0.00 | 0.20 |
| ACACGG | 0.41 | 0.00 | 0.00 | 0.00 | 0.00 | 0.00 | 0.00 | 0.00 | 0.00 | 0.00 | 0.00 | 0.04 |
| ACACTC | 0.23 | 0.16 | 0.00 | 0.00 | 0.00 | 0.00 | 0.00 | 0.00 | 0.00 | 0.00 | 0.00 | 0.04 |
| ACACTG | 0.27 | 0.00 | 0.00 | 0.00 | 0.00 | 0.00 | 0.00 | 0.00 | 0.00 | 0.00 | 0.00 | 0.02 |
| ACAGAG | 3.11 | 1.64 | 0.00 | 0.00 | 0.00 | 0.00 | 0.00 | 0.00 | 0.00 | 0.00 | 0.00 | 0.43 |
| ACAGCC | 0.50 | 0.00 | 0.00 | 0.00 | 0.00 | 0.00 | 0.00 | 0.00 | 0.00 | 0.00 | 0.00 | 0.05 |
| ACAGGC | 2.61 | 0.25 | 0.00 | 0.00 | 0.00 | 0.00 | 0.38 | 0.00 | 0.00 | 0.00 | 0.00 | 0.29 |
| ACAGGG | 0.18 | 0.00 | 0.00 | 0.00 | 0.00 | 0.00 | 0.00 | 0.00 | 0.00 | 0.00 | 0.00 | 0.02 |
| ACAGGT | 0.00 | 0.12 | 0.00 | 0.00 | 0.00 | 0.00 | 0.00 | 0.00 | 0.00 | 0.00 | 0.00 | 0.01 |
| ACAGTC | 3.29 | 0.00 | 0.00 | 0.00 | 0.00 | 0.00 | 0.00 | 0.00 | 0.00 | 0.00 | 0.00 | 0.30 |
| ACAGTG | 0.23 | 0.00 | 0.00 | 0.00 | 0.00 | 0.13 | 0.27 | 0.00 | 0.00 | 0.00 | 0.00 | 0.06 |
| ACCACG | 0.14 | 0.00 | 0.00 | 0.00 | 0.00 | 0.00 | 0.00 | 0.00 | 0.00 | 0.00 | 0.00 | 0.01 |
| ACCAGC | 1.23 | 0.00 | 0.00 | 0.00 | 0.00 | 0.00 | 0.27 | 0.00 | 0.00 | 0.00 | 0.00 | 0.14 |
| ACCCCC | 0.18 | 0.00 | 0.00 | 0.00 | 0.00 | 0.00 | 0.00 | 0.00 | 1.33 | 0.00 | 0.00 | 0.14 |
| ACCCGG | 0.00 | 0.00 | 0.00 | 0.00 | 0.00 | 0.00 | 0.16 | 0.00 | 0.00 | 0.00 | 0.00 | 0.01 |
| ACCCTG | 0.00 | 0.00 | 0.13 | 0.00 | 0.00 | 0.00 | 0.00 | 0.00 | 0.00 | 0.00 | 0.00 | 0.01 |
| ACCGCC | 0.00 | 0.00 | 0.00 | 0.00 | 0.00 | 0.00 | 0.00 | 0.00 | 0.74 | 0.00 | 0.00 | 0.07 |
| ACGAGC | 0.96 | 0.00 | 0.00 | 0.00 | 0.00 | 0.00 | 0.00 | 0.00 | 0.00 | 0.00 | 0.00 | 0.09 |
| ACGAGG | 0.27 | 0.00 | 0.00 | 0.00 | 0.00 | 0.00 | 0.00 | 0.00 | 0.00 | 0.00 | 0.00 | 0.02 |
| ACGCAG | 0.14 | 0.00 | 0.00 | 0.00 | 0.00 | 0.00 | 0.00 | 0.00 | 0.00 | 0.00 | 0.00 | 0.01 |
| ACGGCC | 0.46 | 0.00 | 0.00 | 0.00 | 0.00 | 0.00 | 0.00 | 0.00 | 0.00 | 0.00 | 0.00 | 0.04 |
| ACTACC | 0.55 | 0.57 | 0.00 | 0.00 | 0.61 | 0.61 | 0.27 | 0.00 | 0.00 | 0.00 | 0.00 | 0.24 |
| ACTACG | 0.23 | 0.00 | 0.00 | 0.00 | 0.00 | 0.00 | 0.22 | 0.00 | 0.00 | 0.00 | 0.00 | 0.04 |
| ACTAGG | 0.41 | 0.00 | 0.00 | 0.00 | 0.00 | 0.00 | 0.00 | 0.00 | 0.00 | 0.00 | 0.00 | 0.04 |
| ACTCAG | 0.00 | 0.00 | 0.00 | 0.00 | 0.00 | 0.00 | 0.16 | 0.00 | 0.00 | 0.00 | 0.00 | 0.01 |
| ACTCCT | 0.00 | 0.00 | 0.00 | 0.00 | 0.00 | 0.17 | 0.00 | 0.00 | 0.00 | 0.00 | 0.00 | 0.02 |
| ACTCTC | 0.27 | 0.00 | 0.00 | 0.00 | 0.00 | 0.00 | 0.00 | 0.00 | 0.00 | 0.00 | 0.00 | 0.02 |
| ACTCTG | 0.00 | 0.00 | 0.00 | 0.00 | 0.00 | 0.13 | 0.00 | 0.00 | 0.00 | 0.00 | 0.00 | 0.01 |
| ACTGCT | 1.69 | 0.98 | 0.00 | 0.00 | 0.44 | 0.39 | 0.00 | 0.00 | 0.00 | 0.00 | 0.00 | 0.32 |
| ACTGGT | 0.27 | 0.00 | 0.00 | 0.00 | 0.00 | 0.00 | 0.00 | 0.00 | 0.00 | 0.00 | 0.00 | 0.02 |
| AGAGCG | 0.00 | 0.16 | 0.00 | 0.00 | 0.00 | 0.00 | 0.00 | 0.00 | 0.00 | 0.00 | 0.00 | 0.01 |
| AGAGGC | 0.41 | 0.00 | 0.00 | 0.00 | 0.00 | 0.00 | 0.00 | 0.00 | 0.00 | 0.00 | 0.00 | 0.04 |
| AGAGGG | 1.60 | 0.00 | 0.00 | 0.00 | 0.00 | 0.00 | 0.00 | 0.00 | 1.03 | 0.00 | 0.00 | 0.24 |
| AGCAGG | 0.32 | 0.00 | 0.00 | 0.00 | 0.00 | 0.00 | 0.00 | 0.00 | 0.00 | 0.00 | 0.00 | 0.03 |
| AGCCGG | 0.00 | 0.00 | 0.61 | 0.00 | 0.00 | 0.00 | 0.00 | 0.00 | 0.00 | 0.00 | 0.00 | 0.06 |
| AGGGGG | 0.50 | 0.41 | 0.00 | 0.00 | 0.00 | 0.00 | 0.16 | 0.00 | 2.37 | 0.45 | 0.00 | 0.35 |
| ATACAC | 0.69 | 0.49 | 0.13 | 0.00 | 0.00 | 0.00 | 0.00 | 0.00 | 0.00 | 0.45 | 0.00 | 0.16 |
| ATACAG | 0.00 | 0.12 | 0.13 | 0.00 | 0.00 | 0.00 | 0.00 | 0.00 | 0.00 | 0.00 | 0.00 | 0.02 |
| ATACCG | 0.00 | 0.00 | 0.13 | 0.00 | 0.00 | 0.00 | 0.00 | 0.00 | 0.00 | 0.00 | 0.00 | 0.01 |
| ATACGT | 0.00 | 0.00 | 0.00 | 0.00 | 0.00 | 0.00 | 0.00 | 0.00 | 1.48 | 0.00 | 0.00 | 0.13 |
| ATACTG | 0.00 | 0.00 | 0.00 | 0.00 | 0.00 | 0.00 | 0.22 | 0.00 | 0.00 | 0.00 | 0.00 | 0.02 |
| ATAGTC | 1.55 | 0.00 | 0.00 | 0.00 | 0.00 | 0.00 | 0.00 | 0.00 | 0.00 | 0.00 | 0.00 | 0.14 |
| ATAGGG | 0.00 | 0.12 | 0.00 | 0.00 | 0.00 | 0.00 | 0.00 | 0.00 | 0.00 | 0.00 | 0.00 | 0.01 |
| ATAGTG | 0.14 | 0.00 | 0.00 | 0.00 | 0.00 | 0.13 | 0.00 | 0.00 | 0.89 | 0.00 | 0.00 | 0.11 |
| ATATAC | 0.73 | 0.29 | 0.13 | 0.00 | 0.00 | 0.00 | 0.32 | 0.00 | 0.00 | 1.50 | 0.00 | 0.27 |
| ATATAG | 0.41 | 0.00 | 0.00 | 0.00 | 0.00 | 0.00 | 0.00 | 0.44 | 0.00 | 0.90 | 0.00 | 0.16 |
| **ATCACC** | 1.92 | 2.17 | 0.39 | 0.00 | 0.00 | 0.00 | 2.75 | 0.00 | 4.88 | 0.00 | 0.00 | 1.10 |
| ATCACG | 0.23 | 0.00 | 0.00 | 0.00 | 0.00 | 0.00 | 0.00 | 0.00 | 0.00 | 0.00 | 0.00 | 0.02 |
| ATCAGC | 0.23 | 0.25 | 0.00 | 0.00 | 0.00 | 0.00 | 0.32 | 0.00 | 0.00 | 0.00 | 0.00 | 0.07 |
| ATCATG | 0.00 | 0.00 | 0.00 | 0.00 | 0.00 | 0.00 | 0.16 | 0.00 | 0.00 | 0.00 | 0.00 | 0.01 |
| ATCGCC | 0.00 | 0.12 | 0.17 | 0.00 | 0.00 | 0.00 | 0.22 | 0.00 | 2.37 | 0.00 | 0.00 | 0.26 |
| ATCGGC | 0.00 | 0.00 | 0.00 | 0.00 | 0.00 | 0.00 | 0.00 | 0.15 | 0.00 | 0.00 | 0.00 | 0.01 |
| ATCGTC | 0.59 | 0.74 | 0.26 | 0.00 | 0.17 | 0.00 | 0.22 | 0.00 | 0.74 | 0.00 | 0.00 | 0.25 |
| ATCTAC | 0.69 | 0.49 | 0.13 | 0.00 | 0.50 | 0.13 | 0.16 | 0.00 | 3.55 | 0.00 | 0.00 | 0.51 |
| ATCTGC | 0.14 | 0.00 | 0.00 | 0.00 | 0.00 | 0.00 | 0.00 | 0.00 | 0.00 | 0.00 | 0.00 | 0.01 |
| ATCTGG | 0.00 | 0.00 | 0.00 | 0.00 | 0.00 | 0.00 | 0.16 | 0.00 | 0.00 | 0.00 | 0.00 | 0.01 |
| ATGAGC | 0.00 | 0.20 | 0.00 | 0.00 | 0.00 | 0.00 | 0.00 | 0.00 | 0.00 | 0.00 | 0.00 | 0.02 |
| ATGTAC | 0.27 | 0.00 | 0.00 | 0.00 | 0.00 | 0.00 | 0.00 | 0.00 | 0.00 | 0.00 | 0.00 | 0.02 |
| ATGTCC | 0.00 | 0.20 | 0.00 | 0.00 | 0.00 | 0.00 | 0.00 | 0.00 | 0.00 | 0.00 | 0.00 | 0.02 |
| ATGTGC | 0.32 | 0.16 | 0.00 | 0.00 | 0.00 | 0.00 | 0.00 | 0.00 | 0.00 | 0.00 | 0.00 | 0.04 |

## Table S3 - Results from the BLASTx alignments of Cnidarian sequences and *Symbiodinium* sequences.

| Species | Number of sequences | E-value (high) | E-value (low) | Maximum alignment length |
| --- | --- | --- | --- | --- |
| *Amplexidiscus* | 66 | E-19 | E-54 | 110 |
| *Tanacetipathes* | 247 | E-05 | E-62 | 132 |
| *Corynactis* | 94 | E-19 | E-65 | 108 |
| *Eunicea* | 110 | E-18 | E-63 | 108 |
| *Leiopathes* | 127 | E-17 | E-64 | 112 |
| *Metridium* | 167 | E-19 | E-60 | 103 |
| *Millepora* | 24 | E-23 | E-46 | 83 |
| *Plumarella* | 92 | E-16 | E-53 | 106 |
| Alignment length > 50  Percent ID >75 | | | | |

## Table S4 - Accession numbers of the sequences (700 bp) used to construct the Cytochrome Oxidase I genealogy

| *Species* | Accession number |
| --- | --- |
| *L. glaberrima* | GenBank: FJ597644.1 |
| *Bathypathes sp* | GenBank: GQ200623.1 |
| *E. flexuosa* | GenBank: GQ342445.1 |
| *C. californica* | GenBank: AB441256.1 |
| *A. fenestrafer* | GenBank: AB441267.1 |
| *M. senile* | GenBank: NC000933.1 |
| *M. alcicornis* | GenBank: KC570602.1 |
| *N. vectensis* | GenBank: DQ643835.1 |
| *H. magnipapillata* | GenBank: AB565139.1 |
| *A. digitifera* | GenBank: AF338425.1 |

## Table S5 - Average microsatellite length (average nucleotide length/microsatellite type) found in Cnidaria

| Length (nt average length/type) | *Leiopathes* | *Tanacetipathes* | *Eunicea* | *Plumarella* | *Corynactis* | *Amplexidiscus* | *Metridium* | *Millepora* | *Nematostella* | *Hydra* | *Acropora* | Mean |
| --- | --- | --- | --- | --- | --- | --- | --- | --- | --- | --- | --- | --- |
| Mono- | 20.17 | 18.98 | 17.71 | 23.56 | 17.90 | 21.41 | 19.69 | 20.45 | 23.97 | 25.16 | 85.64 | 26.79 |
| Di- | 23.28 | 18.14 | 11.55 | 14.55 | 12.03 | 12.94 | 15.81 | 17.20 | 25.80 | 39.39 | 33.86 | 20.41 |
| Tri- | 12.63 | 14.86 | 16.28 | 11.68 | 11.31 | 11.53 | 11.14 | 13.61 | 18.19 | 15.35 | 14.65 | 13.75 |
| Tetra- | 8.32 | 10.51 | 9.62 | 16.38 | 6.66 | 5.88 | 10.93 | 17.65 | 16.30 | 11.83 | 17.62 | 11.97 |
| Penta- | 5.75 | 5.48 | 5.09 | 7.76 | 4.54 | 3.76 | 7.15 | 4.13 | 9.80 | 3.67 | 21.08 | 7.11 |
| Hexa- | 8.27 | 7.27 | 7.24 | 7.58 | 11.04 | 9.25 | 8.37 | 4.11 | 9.10 | 4.24 | 4.73 | 7.38 |
| Mean | 13.07 | 12.54 | 11.25 | 13.59 | 10.58 | 10.79 | 12.18 | 12.86 | 17.19 | 16.61 | 29.60 | 14.57 |

## Table S6 - Mann-Whitney Rank Sum Test between microsatellite cover (A) and microsatellite length (B) of symbiotic and non-symbiotic Cnidarians

N = the mean of microsatellite coverage for the species. Included the number of missing values, the median and the 25 and 75 % quartiles of each group.

A)

| Group | N | Missing | Median | 25% | 75% |
| --- | --- | --- | --- | --- | --- |
| Non-Symbiotic | 30 | 0 | 45.89 | 14.14 | 91.38 |
| Symbiotic | 18 | 0 | 14.74 | 7.53 | 22.77 |
| Mann-Whitney U Statistic = 125.00 | | |  |  |  |
| T = 296.00, n_small_ = 18, n_big_ = 30, p = 0.002 | | | |  |  |

B)

| Group | N | Missing | Median | 25% | 75% |
| --- | --- | --- | --- | --- | --- |
| Non-Symbiotic | 30 | 0 | 11.23 | 7.72 | 16.76 |
| Symbiotic | 18 | 0 | 11.54 | 5.68 | 17.31 |
| Mann-Whitney U Statistic = 248.00 | | |  |  |  |
| T = 419.00, n_small_ = 18, n_big_ = 30, p = 0.647 | | | |  |  |

## Table S7 - Mann-Whitney Rank Sum Test between microsatellite cover (A) and microsatellite length (B) for species with fast or slow mitochondrial evolution

N = the mean of microsatellite coverage for the species. Displayed the number of missing values, the median and the 25 and 75% quartiles of each group.

A)

| Group | N | Missing | Median | 25% | 75% |
| --- | --- | --- | --- | --- | --- |
| Slow | 54 | 0 | 43.48 | 14.49 | 103.95 |
| Fast | 12 | 0 | 27.15 | 12.50 | 82.15 |
| Mann-Whitney U Statistic = 293.00 | | |  |  |  |
| T = 371.00, n_small_ = 12, n_big_ = 54, p = 0.61 | | | |  |  |

B)

| Group | N | Missing | Median | 25% | 75% |
| --- | --- | --- | --- | --- | --- |
| Slow | 54 | 0 | 11.62 | 8.14 | 17.96 |
| Fast | 12 | 0 | 14.48 | 4.16 | 19.75 |
| Mann-Whitney U Statistic = 323.000 | | |  |  |  |
| T = 401.00, n_small_ = 12 n_big_ = 54, p = 0.99 | | | |  |  |
