# Supplementary material for: Microsatellite abundance across the Anthozoa and Hydrozoa in the phylum Cnidaria
Source: BMC Genomics. 2014 Oct 27;15(1):939. doi: 10.1186/1471-2164-15-939 (PMC4226868; doi:10.1186/1471-2164-15-939)
Supplement: Supplementary file 2 — Additional file 2: Summary statistics. (DOCX 121 KB) [file 12864_2013_6637_MOESM2_ESM.docx]

### GC content

Mean GC content ranged from 33% to 43% (Figure 2) in the 8 partial genome sequences (PGS) and from 23% to 42% in the WGS species. GC content differed between the PGS and the WGS (2 tailed t-test, p < 0.001). GC content also differed among PGS species (Kruskal-Wallis One Way ANOVA, p < 0.005) and among WGS species (Kruskal-Wallis One Way ANOVA, p < 0.001). For within PGS comparisons, the GC content was significantly different between *Leiopathes* and *Millepora*. Of the WGS species, *Nematostella* had higher GC content then *Hydra* and *Acropora* (Figure 2).

### Microsatellite coverage

When comparing the microsatellite coverage (total microsatellite bases within a megabase of sequence) in PGS and WGS, we found clear differences in some microsatellite types while others were similar across sequencing methodologies (Figure 3). The observed microsatellite coverage was similar between PGS and WGS species when considering trinucleotides (t-test, p = 0.08), tetra- (t-test, p=0.21), pentanucleotides (t-test, p=0.37) and hexanucleotides (t-test, p = 0.36), but differed for mono- (t-test, p = 0.01) and dinucleotides (t-test, p = 0.01) (Figure 3, Table S1).

All microsatellite types were found in all species (mono-, di-, tri-, tetra-, penta- and hexanucleotides), with the exception of *C. californica* in which mononucleotides were not detected. Of all possible motifs, we found 2 motifs of mono-, 4 di-, 10 tri-, 33 tetra-, 77 penta-, and 160 types of hexanucleotides. Overall, trinucleotides and tetranucleotides were the most abundant types in Cnidaria, but noticeable differences among species were observed (Figure 3).

Mononucleotides were rare in the PGS species (coverage varied from 4 to 11), but under-representation of mononucleotides might be due to difficulties of sequencing mononucleotides with *454* [[20](#_ENREF_20)]. However, mononucleotide coverage was high in species with complete genomes (Table 2, Figure 3).

Dinucleotides were frequent in the hydrozoan *H.* *magnipapillata*, the anemone *N. vectensis*, the coral *A. digitifera* and the antipatharian *L. glaberrima*, but rare in the other species (coverage ≤ 40) (Table 2, Figure 3) (Kruskal-Wallis One Way ANOVA, p = 0.02). The motifs AC, AT and AG were common, while GC was rare in all species (Table 3). AC was the only dinucleotide motif in *A. fenestrafer*.

Trinucleotide coverage was higher in black corals (*Leiopathes*, *Tanacetipathes*), anemones (*Metridium*, *Nematostella*), and *Acropora* (Table 2, Figure 3) than in the other species. Of the PGS species, trinucleotide coverage in *Leiopathes* was significantly different from *Millepora* and *Corynactis* (Kruskal-Wallis One Way ANOVA, p = 0.001). No difference was observed among the WGS species. AAT, ATC, AAC and ACT were the most abundant trinucleotide motifs for all cnidarian species (Table 4).

Significant differences in tetranucleotide coverage were observed among *Leiopathes* and *Eunicea*, *Millepora* and the Corallimorpharians, and among *Metridium*, *Eunicea* and *Amplexidiscus* (Kruskal-Wallis One Way ANOVA, p = 0.001). *Nematostella* also differed from *Hydra* and *Acropora* (Kruskal-Wallis One Way ANOVA, p = 0.001). ATAC, ATAG, AAAT, ACAG and AAAC were the most abundant tetranucleotide motifs (Table 5).

In the PGS data set, pentanucleotide coverage varied from 17 to 85 (Table 2 B, Figure 3). The motifs AAAAT, AATAC and AAAAC were the most common pentanucleotides in all species (PGS and WGS) (Additional file 1: Table S2). AATAT coverage was highest in *Acropora*, and AGAGC was dominant in the anthipatharians and *Metridium*. *Eunicea* and *Amplexidiscus* have significantly less pentanucleotide motifs than *Leiopathes* and *Metridium* (Kruskal-Wallis One Way ANOVA, p = 0.001). *Acropora* has higher motif coverage than the other two WGS species (Kruskal-Wallis One Way ANOVA, p = 0.003).

*Plumarella spp*., *Leiopathes* and *Acropora* had the highest coverage of hexanucleotides (Table 2 B, Figure 3). *Plumarella* and *Leiopathes* were significantly different from the other PGS species (Kruskal-Wallis One Way ANOVA, p = 0.001). Of the WGS species, *Nematostella* and *Acropora* differed in their hexanucleotide coverage (Kruskal-Wallis One Way ANOVA, p = 0.001). The motif type AACCCT was by far the most frequent hexanucleotide motif in the Cnidaria (Additional file 1: Table S1).

### Microsatellite lengths (number of repeat units)

Analysis of Variances (ANOVA) of the microsatellite lengths suggested significant differences (p<0.004) among species for the length of all microsatellite types (Figure 4). Mean repeat numbers ranged from 4 to 23 (repeats/microsatellite type) for all microsatellites types in all PGS species and between 4 and 86 repeats for the microsatellite types in the WGS species. However, sequences with long microsatellites (e.g. > 23 repeats) were rare (mean of means=14.6 repeats, s.d.=5.4) in both WGS and PGS.

The absolute number of mononucleotide repeats was highly variable in *Plumarella spp, Metridium* and *Acropora.* The mean length for mononucleotides was 23 repeats (s.d.= 14.6). Mononucleotide lengths differed among PGS species (Kruskal-Wallis One Way ANOVA, p = 0.004), but not among WGS species (Kruskal-Wallis One Way ANOVA, p = 0.98, Figure 4).

Most frequent dinucleotide lengths were between 12 and 39 repeats with means of 31.8 repeats (s.d.= 26.3). *Leiopathes* and *Hydra* had the longest dinucleotide repeats: 183 repeats in *Leiopathes* and 402 repeats in *Hydra*. The dinucleotide lengths of *Leiopathes*, *Tanacetipathes* and *Millepora* were significantly different from the other PGS species (Kruskal-Wallis One Way ANOVA, p = 0.001), while all WGS species were significantly different from each other (Kruskal-Wallis One Way ANOVA, p = 0.001, Figure 4).

The majority of the trinucleotides had between 11 and 18 repeats, and mean length of 13.6 repetitions (s.d.=12.9). Of the WGS species*, Acropora* presented the longest trinucleotides (189 repetitions), and *Nematostella* and *Hydra* were significantly different in their trinucleotide lengths (Kruskal-Wallis One Way ANOVA, p = 0.001, Figure 4). In the PGS species, trinucleotide length was similar for *Leiopathes* and *Eunicea*, and *Corynactis* and *Millepora* (Kruskal-Wallis One Way ANOVA, p = 0.001, Figure 4).

Repeat lengths of tetranucleotide was between 3.75 and 186.25 repetitions with mean length of 11.4 repeats (s.d.=12.3). Sequences with a high number of tetranucleotide repeats were found in *Tanacetipathes spp,* *Metridium,* *M. alcicornis* and all WGS species (Figure 4). Tetranucleotide lengths differ among all PGS species, except *Tanacetipathes*, *Eunicea* and *Metridium* that had similar variances. As in trinucleotides, the tetranucleotide lengths differed between *Nematostella* and *Hydra* (Kruskal-Wallis One Way ANOVA, p = 0.001, Figure 4).

The repeat length of pentanucleotide was between 3 to 150 repeats with mean of 6.1 repeats (s.d= 7.8). The longest pentanucleotide repeats were found in *Metridium*, *Plumarella* and *Acropora*. Pentanucleotide lengths differed for most of the PGS species (Kruskal-Wallis One Way ANOVA, p = 0.001, Figure 4). While length of pentanucleotides in *Hydra* were different from the lengths in *Nematostella* and *Acropora* (Kruskal-Wallis One Way ANOVA, p = 0.001, Figure 4).

In hexanucleotides, the absolute number of repeats varied from 3 to 89.7, the mean length was 7.9 (s.d.= 7.4). Lengths differed among most species, but *Plumarella*, *Corynactis* and *Millepora*, *Metridium* and *Eunicea* had similar lengths (Kruskal-Wallis One Way ANOVA, p = 0.001, Figure 4). The hexanucleotide lengths in *Hydra* were different than those of *Nematostella* and *Acropora* (Kruskal-Wallis One Way ANOVA, p = 0.001, Figure 4).

## Tables

## Table S1 - Mann-Whitney U statistics (A) and t-test results (B) between microsatellites coverage of PGS and WGS species

N = the number of species, Missing = the number of missing values, Median = the median of the microsatellite coverage, 25 and 75% are quartiles of each group, U = U statistic, T = Mann-Whitney T statistic, p = p-value, Mean = the mean of the microsatellite coverage, Std Dev = Standard deviation, SEM = Standard error of the mean, Difference = difference in the mean values, t = t statistic, df = degrees of freedom.

A.

| Group | N | Missing | Median | 25% | 75% | U | T | p |  |
| --- | --- | --- | --- | --- | --- | --- | --- | --- | --- |
| Dinucleotides PGS | 8 | 0 | 10.12 | 5.192 | 39.12 | 0 | 30 | 0.01 |  |
| Dinucleotide WGS | 3 | 0 | 230.8 | 198.22 | 1249.22 |  |  |  |  |
| Trinucleotide PGS | 8 | 0 | 42.22 | 22.345 | 137.21 | 3 | 27 | 0.08 |  |
| Trinucleotide WGS | 3 | 0 | 353.73 | 76.88 | 594.02 |  |  |  |  |
| B. |  |  |  |  |  |  |  |  |  |
| Group Name | N | Missing | Mean | Std Dev | SEM | Difference | t | df | p |
| Tetranucleotide PGS | 8 | 0 | 90.41 | 74.69 | 26.41 | -69.30 | -1.35 | 9 | 0.21 |
| Tetranucleotide WGS | 3 | 0 | 159.72 | 80.32 | 46.38 |  |  |  |  |
| Pentanucleotide PGS | 8 | 0 | 40.99 | 25.83 | 9.13 | -17.14 | 0.94 | 9 | 0.37 |
| Pentanucleotide WGS | 3 | 0 | 58.14 | 29.52 | 17.05 |  |  |  |  |
| Hexanucleotide PGS | 8 | 0 | 37.56 | 33.57 | 11.87 | -23.19 | -0.97 | 9 | 0.36 |
| Hexanucleotide WGS | 3 | 0 | 60.74 | 40.63 | 23.46 |  |  |  |  |
